# Enhanced Charge Transport through Ion Networks in Highly Concentrated LiSCN‐Polyethylene Carbonate Solid Polymer Electrolytes

**DOI:** 10.1002/smsc.202400653

**Published:** 2025-01-25

**Authors:** Kajal Kumbhakar, Sourav Palchowdhury, Thuy Duong Pham, Seoeun Shin, So Yeon Chun, Joong Won Shim, Kyung‐Koo Lee, Minhaeng Cho, Kyungwon Kwak

**Affiliations:** ^1^ Center for Molecular Spectroscopy and Dynamics Institute for Basic Science (IBS) Seoul 02841 Republic of Korea; ^2^ Faculty of Biotechnology, Chemistry and Environmental Engineering Phenikaa University Hanoi 10000 Vietnam; ^3^ Department of Chemistry Korea University Seoul 02841 Republic of Korea; ^4^ Department of Chemistry Kunsan National University Gunsan Jeonbuk 54150 Republic of Korea

**Keywords:** enhanced ion transports, ion channels, IR spectroscopy, polymer‐in‐salt electrolytes, weak cation‐polymer interactions

## Abstract

Challenging the preference for bulky anions due to low binding energy with Li^+^ ion, the lithium thiocyanate‐polyethylene carbonate (LiSCN‐PEC) solid polymer electrolyte (SPE) demonstrates higher ionic conductivities (3.16 × 10^−5^ S cm^−1^) at polymer‐in‐salt concentration (100 mol%) compared to those with lithium bis(fluorosulfonyl)imide (LiFSI, 1.01 × 10^−5^ S cm^−1^) and lithium bis(trifluoromethanesulfonyl)imide (LiTFSI, 1.72 × 10^−7^ S cm^−1^). Through the careful selection of PEC and LiSCN as components of SPE, the carbonyl stretching of PEC and the SCN^−^ stretching band as vibrational reporters provide detailed structural insights into the Li^+^ ion transport channel. Spectroscopic investigations reveal that enhanced ion aggregation alters the solvation structure around the Li^+^ and diminishes the interaction between Li^+^ and polymer (PEC) with increasing LiSCN concentrations, promoting faster segmental motion as a major transport mechanism. However, the transition observed from subionic to superionic behavior in the Walden plot indicates the onset of segmental motion decoupled charge transport pathway. The SCN^−^ vibrational spectrum elucidates the evolution from a Li–SCN–Li type chain‐like structure to a Li_2_ > SCN < Li_2_ type extended ion network with increasing LiSCN concentration, revealing that the ion network provides an alternative channel for Li^+^ ion transfer at higher concentrations, enhancing conductivity.

## Graphical abstract

The significantly enhanced ion conductivity in highly concentrated solid polymer electrolytes which is 100 mol% LiSCN (lithium thiocyanate) in polymer PEC (polyethylene carbonate) here is a manifestation of combined effects arising from Li‐ion transport through polymer backbone with faster polymer segmental motion and additional faster Li‐ion transport through percolated ion network channel.

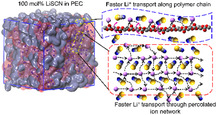

## 1 Introduction

Solid polymer electrolytes (SPEs) are promising for high energy density rechargeable Li‐ion batteries (LIBs) due to high mechanical, thermal, and electrochemical stability.^[^ ^1^ ^]^ Since the discovery of SPEs based on polyethylene oxide (PEO),^[^ ^2^ ^]^ there have been various efforts to overcome their low ionic conductivity (*σ*_dc_ < 10^−3^ S cm^−1^). However, low conductivity and Li^+^‐ion transference number (*t*_+_) still limit the potential of polyether‐based SPEs.

Since the discovery of polymer‐in‐salt (PIS) SPEs,^[^ ^3^ ^]^ highly Li‐salt concentrated SPEs have provided a new avenue to improve *σ*_dc_ and *t*_+_ of SPEs. Research indicates that, in addition to ion diffusion facilitated by polymer segmental motion in salt‐in‐polymer (SIP) SPEs, highly concentrated PIS SPEs exhibit additional ion transport pathways, enabling high ionic conductivity. For example, a cross‐linked Li^+^ transportation channel was proposed in poly(vinylidene fluoride‐co‐hexafluoropropylene)/LiFSI/LLZTO composite PIS electrolytes to explain its high conductivity of 1.67 × 10^−3^ S cm^−1^.^[^ ^4^ ^]^ Molecular dynamics (MD) simulation studies reported that metal ion diffusion through molten salt region causes a decoupling of conductivity from polymer segmental motion in polymeric ionic liquid‐based PIS SPEs.^[^ ^5^ ^]^ The percolation phenomena for ion conduction were also postulated to explain the negligible influence of polymer segmental motion on ionic conductivity enhancement in PAN/LiCF_3_SO_3_ PIS electrolytes.^[^ ^6^ ^]^

Polyethylene carbonate (PEC), owing to its capability to dissolve large amounts of Li‐salt, can form highly concentrated SPEs with enhanced *σ*_dc_ (>10^−5^ S cm^−1^).^[^ ^7^ ^]^ Additionally, PEC‐Li salt‐based SPEs exhibit higher *t*_+_ than polyether‐based ones, with a linear correlation between *t*_+_ and the anion size.^[^ ^8^ ^]^ However, despite studies on ion transport behavior in PEC‐based SPEs, the microscopic mechanisms behind the influence of anion identity on charge transport behavior in these highly concentrated SPEs remain unclear.^[^ ^9^ ^]^

Here, we developed a lithium thiocyanate (LiSCN)‐PEC SPE with increasing conductivity up to 80 mol% LiSCN. SCN^−^ has not been used before due to concerns that its concentrated charge density may prevent Li^+^ dissociation and restrict Li^+^ ion transfer. However, LiSCN‐PEC exhibits higher conductivity than PEC‐LiTFSI and PEC‐LiFSI with bulky anions. By employing fourier transform infrared (FTIR) spectroscopy analyses on the C=O stretch mode of PEC and the C≡N stretch mode of SCN^−^ along with dielectric relaxation spectroscopy (DRS) and computer simulations, we have investigated Li^+^‐polymer, Li^+^‐anion interactions, Li^+^‐solvation structure, and polymer segmental motion, which reveals that the Li^+^ transport through ion network^[^ ^10^ ^]^ is facilitated with the increasing salt concentration and enhances conductivity in PIS SPEs.

## 2 Results and Discussion

### 2.1 Enhanced Ionic Conductivity of PEC‐Based SPEs with Increasing LiSCN Concentration

**Figure 1:**
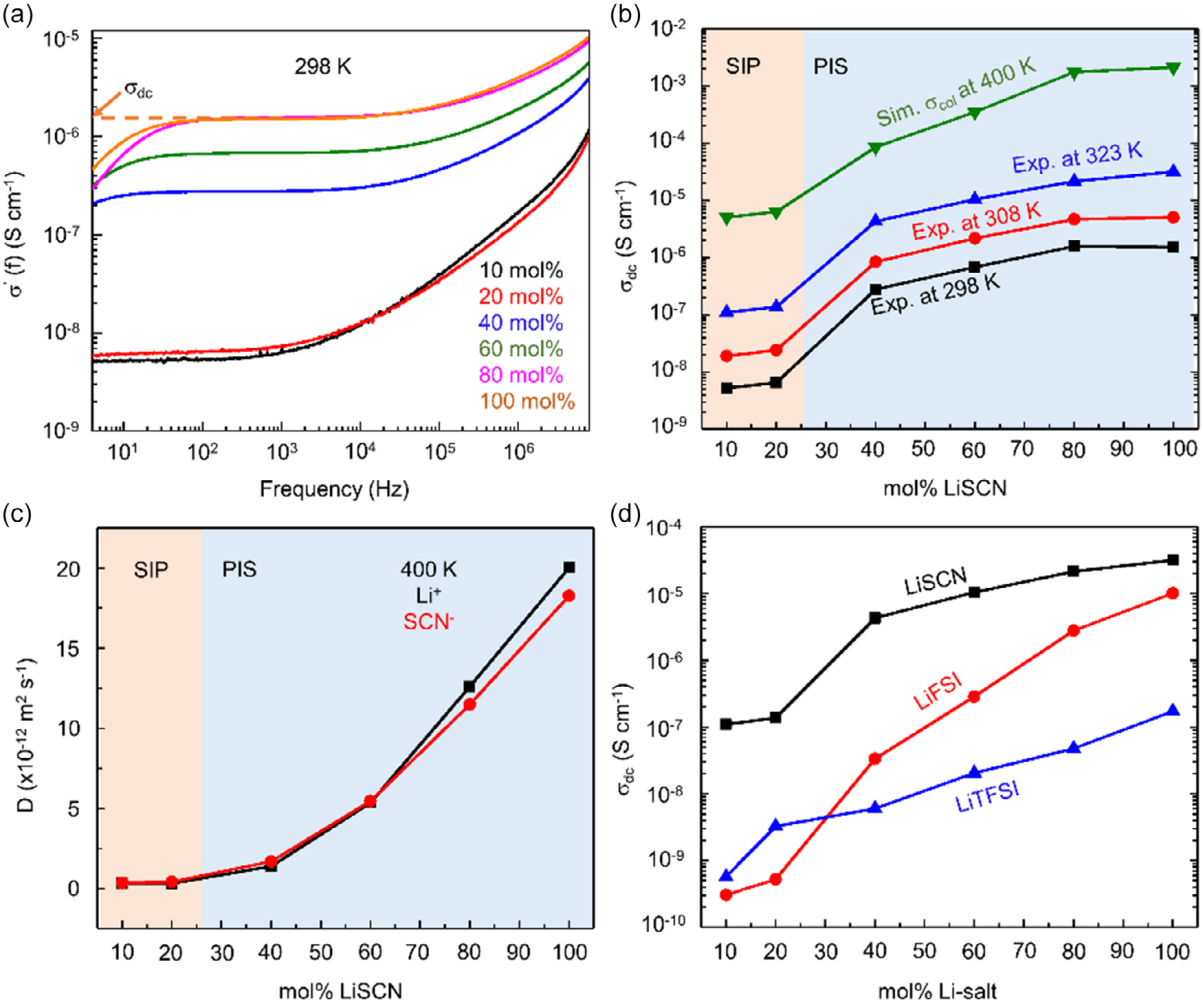
Ionic conductivity enhancement with LiSCN concentration. a) Real part of the experiment complex conductivity spectra in LiSCN‐PEC with varying LiSCN mol% at 298 K. The dashed line represents the extrapolation of frequency independent plateau region to frequency, *f* = 0 to get DC ionic conductivity (*σ*_dc_). b) LiSCN concentration‐dependent variation of *σ*_dc_ measured experimentally and *σ*_col_ calculated from MD simulation. c) Translational self‐diffusion coefficients of Li^+^ and SCN^−^ ions. SIP and PIS region in Figure 1b,c are indicated by different color shades. d) Comparison of experimental ionic conductivity between LiSCN‐PEC, LiFSI‐PEC, and LiTFSI‐PEC SPEs.

Ionic conductivities (*σ*_dc_) extracted (Note S2, Supporting Information) from the frequency‐dependent real conductivity spectra (*σ*′(f)) in **Figure** **1**a rise little till 20 mol% LiSCN and then show a significant enhancement beyond 20 mol% up to 80 mol% as shown in Figure 1b. At higher temperatures also, from 298 to 323 K, *σ*_dc_ shows a monotonous increase with the same concentration dependence, indicating a transition of conductivity at >20 mol% emerges mainly from switching the SIP regime to PIS. It confirms our analysis at 298 K represents the ion conduction behavior at 323 K which is close to temperature in working battery.

MD simulations were performed on LiSCN‐PEC SPEs spanning the entire concentration range probed by the experiment (see detailed simulation methodology and system setup protocols in the Note S3, Supporting Information). The translational self‐diffusion coefficient has been calculated from the slope of the diffusive regime of the mean‐squared displacement (MSD) plot (Note S4 and Figure S3a, Supporting Information). We have observed an increase in the amplitude and slope of the MSDs for both Li^+^ and SCN^−^ ions with increasing LiSCN mol% in the SPEs simulated at 400 K. The calculated self‐diffusion coefficients for Li^+^ (D^+^) and SCN^−^ (D^−^) increase with higher salt concentration at 400 K (Figure 1c). This contrasts with the usual diffusion behavior of conventional electrolyte solutions where translational mobilities of ions diminish with increasing salt concentration. The diffusion coefficients of Li^+^ and SCN^−^ are quite close at a given salt concentration, indicating a collective ion motion. Therefore, we computed ionic conductivities (*σ*_col_) accounting for correlated ion motions using the Einstein form of the equation (see Note S4, Supporting Information)^[^ ^11^ ^]^ at 400 K, which exhibits a similar LiSCN concentration dependence with the experimental one at lower temperatures, though much higher magnitude as expected from temperature dependence shown in Figure 1b. The *σ*_NE_ is calculated from the self‐diffusion coefficient of D^+^ and D^−^ according to Nernst–Einstein equation (Note S4, Supporting Information). The computed *σ*_NE_ shows a similar trend to *σ*_col_ with salt concentration (Figure S3b, Supporting Information), though higher in magnitude as it does not consider collective ionic movements.

LiSCN‐PEC SPEs yield higher ionic conductivity than LiTFSI‐PEC and LiFSI‐PEC SPEs. This experimental (Figure 1d) and simulation (Figure S4a, Supporting Information) results are surprising considering the conventional notion that electrolytes with bulky anions exhibit better conductivity due to reduced charge density and resulting low binding energy with Li^+^ in both low and highly concentrated electrolytes. The justification behind the higher conductivity of LiSCN‐PEC SPEs over LiFSI‐PEC and LiTFSI‐PEC SPEs is explained in Note S5, Supporting Information. This necessitates reconsidering the anion selection criteria in highly concentrated PIS electrolytes and investigating the cation transport mechanism in these SPEs. In the following section, the role of polymer segmental motion on the ion transport mechanism will be elucidated by employing dielectric relaxation spectroscopy and MD simulation.

### 2.2 Faster Polymer Segmental Motion and Decoupled Conductivities with Increasing LiSCN Concentration

**Figure 2:**
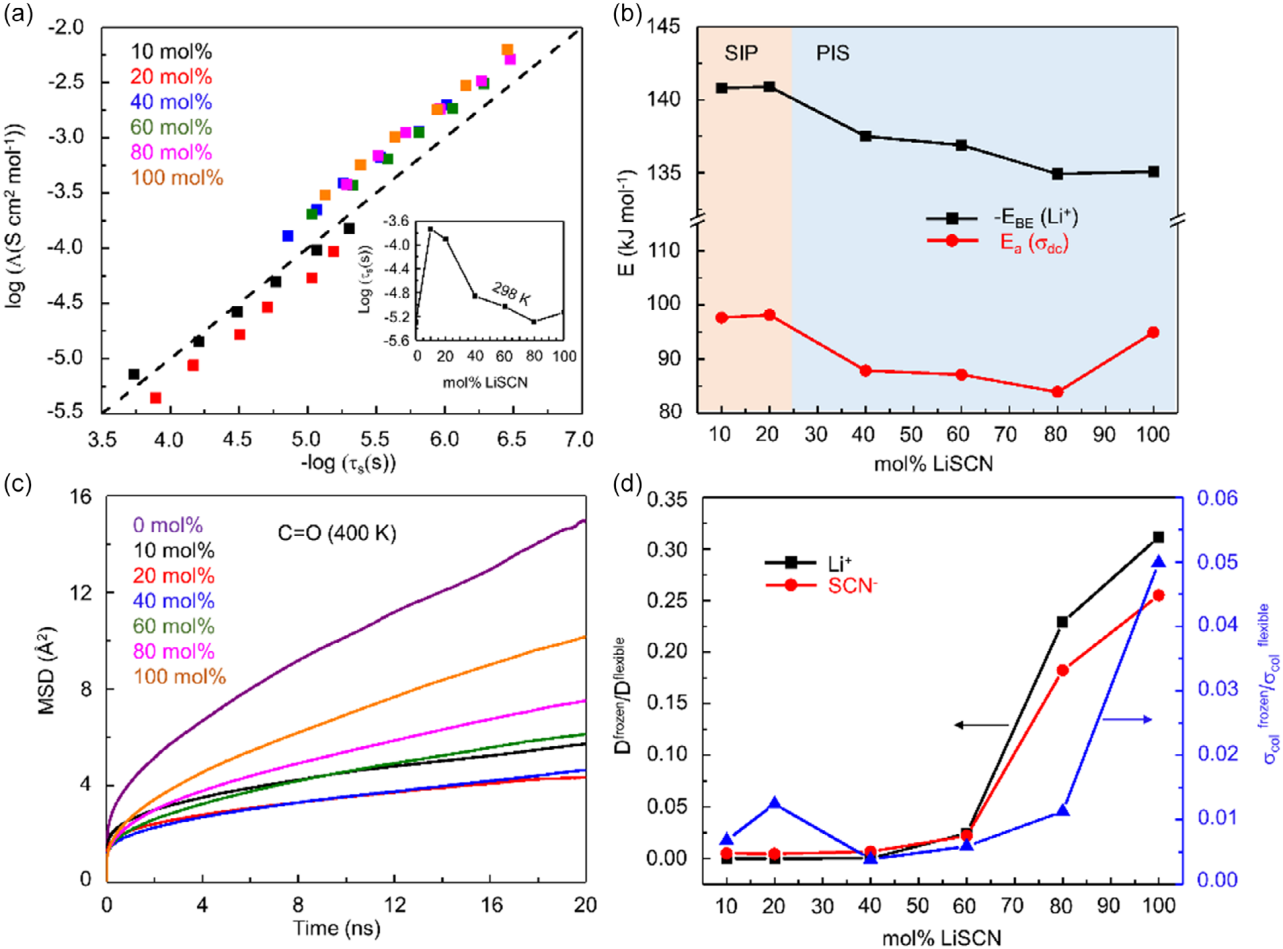
Polymer segmental motion study. a) Walden type plot in LiSCN‐PEC SPE films. Dashed line indicates the ideal Walden line with slope 1. Inset: LiSCN concentration‐dependent variation of *τ*_s_ at 298 K. b) Activation energy associated with experimental *σ*_dc_ (red) and MD simulated average binding energy per Li^+^ ion with the system bath (black) at 400 K. c) MSDs of carbonyl oxygen in the COM frame of the PEC chain. d) The degree of coupling for a dynamic observable <*A*> (D and *σ*_col_) in terms of the ratio of that observable in frozen polymer to flexible polymer simulations (<*A*>^frozen^/<*A*>^flexible^) in LiSCN‐PEC SPEs.

The inset of **Figure** **2**a represents the polymer segmental relaxation times (*τ*_s_) with increasing LiSCN mol% at 298 K (detailed analyses in Note S2, Supporting Information). The addition of 10 mol% LiSCN results in a dramatic increase in *τ*_s_ from 5.06 to 184.65 μs. Subsequently, *τ*_s_ gradually decreases with a further increase in LiSCN mol%, indicating the weakening of interactions between polymers. This is also reflected in the gradual decrease in glass transition temperature (*T*_g_) as shown in Figure S6, Supporting Information.

Activation energies for *σ*_dc_, $E_{a}^{\sigma_{\text{dc}}}$ (Note S6, Supporting Information), show opposite concentration dependences at high and low concentrations (Figure 2b), indicating different ion transport mechanisms between the SIP and PIS SPEs. At lower concentrations, LiSCN enhances interactions between polymer chains like PEO SPEs but acts as plasticizers, increasing the fluidity of SPEs beyond 20 mol% at the PIS region. Notably $E_{a}^{\sigma_{\text{dc}}}$ increases from 80 to 100 mol%, likely indicating another transition in the transport mechanism. The Walden plot, a correlation chart between viscosity and conductivity, has been used to investigate the transport mechanism. In SPEs, molar ionic conductivity ($\Lambda =\frac{\sigma_{\text{dc}}}{c}$) (*c* is molar concentration in unit mol cm^−3^; see Note S7, Supporting Information) is more closely related to *τ*_s_ rather than medium bulk viscosity. Thus, Λ plotted with respect to 1/*τ*_s_ in Figure 2a clearly shows a mild deviation from the ideal Walden line (complete coupling) with slope 1. Furthermore, we observe a transition from a subionic (below ideal line) to a superionic (above ideal line) regime with increasing LiSCN concentration. This deviation with increasing LiSCN concentration indicates that polymer segmental motion is not the sole mechanism for enhancing ionic conductivity in these LiSCN‐PEC SPEs.

The polymer segmental motion can be modeled from the computed MSD of the center of mass (COM) of individual monomer units (see Scheme S1c, Supporting Information) in the COM frame of the polymer chain to which the monomers are attached.^[^ ^12^ ^]^ For PEC, the COM of the monomer unit coincides with the carbonyl carbon atom of the carbonate group. However, the carbonyl oxygen, which anchors the strongest interaction with the Li^+^ ions, can execute additional degrees of freedom compared to the COM of the monomer unit. Therefore, we primarily focus on the MSD of carbonyl oxygen atoms of the PEC chain. We also computed the MSDs of monomers (Note S8, Supporting Information). Figure 2c shows the computed MSDs of carbonyl oxygen atoms across the concentration range at 400 K. The addition of 10 mol% LiSCN to PEC arrests the motion of carbonyl oxygens due to strong binding with Li^+^ ions, resulting in a decrease in MSDs. At 20 mol%, the MSD becomes even smaller, as observed in PEO SPEs. However, MSDs gradually increase beyond 20 mol% onward, surpassing those of 10 mol% from 60 mol%. In addition to the MSDs, the calculated translational diffusion coefficients of carbonyl oxygen atoms (Table S6, Supporting Information), Rouse time associated with the MSDs of monomer (*τ*_R_) (Note S8 and Table S6, Supporting Information), and O‐C‐C‐O dihedral relaxation kinetics (Note S9, Supporting Information) qualitatively follow concentration‐dependent polymer segmental relaxation time, confirming that our MD simulation accurately captures carbonate polymer dynamics.

To estimate the contribution of segmental motion to conductivity, we computed a dynamic observable <*A*> (where *A* is D^+/−^ or *σ*_col_) in frozen and flexible polymer environments (<*A*>^frozen^ and <*A*>^flexible^). As shown in Figure 2d, the translational diffusion coefficients of both ions are significantly coupled to polymer segmental motion up to 40 mol% LiSCN concentration, resulting in *D* ^frozen^/*D* ^flexible^ values close to zero. Beyond this concentration, the ratio steadily increases, reaching ≈0.3 at 100 mol%, indicating a significant decoupling of ion motions from polymer segmental motions at high salt concentrations. When inspecting simulated ionic conductivities, $\sigma_{\text{col}}^{\text{frozen}}/\sigma_{\text{col}}^{\text{felxible}}$ ratio is close to zero till 40 mol% LiSCN concentration and increases to a limiting value of 0.03 for 100 mol%, although *D* ^frozen^/*D* ^flexible^ reached 0.3. These observations suggest that while the individual ions exhibit significant decoupling from polymer segmental motion, the coupling between cations and anions restricts the overall contribution of ion motion to conductivity. This results in a weak superionic nature of the SPEs, as shown in the Walden plot.

### 2.3 Reduced Li^+^ Binding Energies with Increasing LiSCN Concentrations

Figure 2b shows that the average binding energy per cation ($E_{\text{BE}}^{{\text{Li}}^{+}}$) with surrounding molecules (average potential energy arises from nonbonded Coulombic and Lennard‐Jones contributions) decreases with increasing salt concentration. Smaller interaction strength of Li^+^ ion with the environment results in greater mobility and higher ionic conductivity. We further decompose $E_{\text{BE}}^{{\text{Li}}^{+}}$ into ionic and polymer contributions and listed them in **Table** **1**.

**Table 1:** Decomposition of average binding energy and fraction of Li^+^ ($f_{{\text{Li}}^{+}}$) ions bound to carbonyl oxygen at 400 K.

[mol%] LiSCN	$E_{\text{BE}}^{{\text{Li}}^{+}}$[kJ mol^−1^]			$f_{{\text{Li}}^{+}}$
	Total	Ionic	Polymer	
10%	−140.804	−102.822	−37.982	0.98
20%	−140.897	−101.717	−39.180	0.95
40%	−137.495	−106.529	−30.966	0.86
60%	−136.892	115.158	−21.734	0.75
80%	−134.925	−116.323	−18.602	0.65
100%	−135.089	−120.552	−14.537	0.57

Although $E_{\text{BE}}^{{\text{Li}}^{+}}$ decreases with increasing LiSCN mol%, the ionic contribution to $E_{\text{BE}}^{{\text{Li}}^{+}}$ increases, consistent with the formation of an extensive ion network. The contribution of the polymer matrix to the $E_{\text{BE}}^{{\text{Li}}^{+}}$ decreases with increasing salt concentration, indicating a weakening of Li^+^‐polymer interaction. The fraction of Li^+^ bound to carbonyl oxygen ($f_{{\text{Li}}^{+}}$, calculated based on first coordination shell cutoff of *g*(r) of Li–O=C, Table 1) also decreases with increasing salt concentration. Thus, we conclude that increasing LiSCN concentration enhances ionic conductivity due to the weakening of Li^+^‐polymer interaction, resulting in a larger fraction of labile Li^+^ ions decoupled from the polymer. This assumes faster ion transfer when decoupled from polymer segmental motion. To validate this, we simulated pure amorphous LiSCN at 573 K (just above its melting temperature) and compared the calculated ionic conductivity with that of 100 mol% LiSCN‐PEC SPE at the same temperature. Pure LiSCN shows eightfold higher conductivity (0.93 S cm^−1^) than 100 mol% LiSCN‐PEC (0.12 S cm^−1^), where a significant fraction of Li^+^ ions are still bound to the PEC backbone. The faster ion transport of Li^+^ ion decoupled from the polymer chain prompts us to investigate the solvation structural change surrounding Li^+^ in LiSCN PEC SPEs with varying LiSCN concentration.

### 2.4 Li^+^‐Polymer Interaction Becomes Weaker with Increasing LiSCN Concentrations

**Figure 3:**
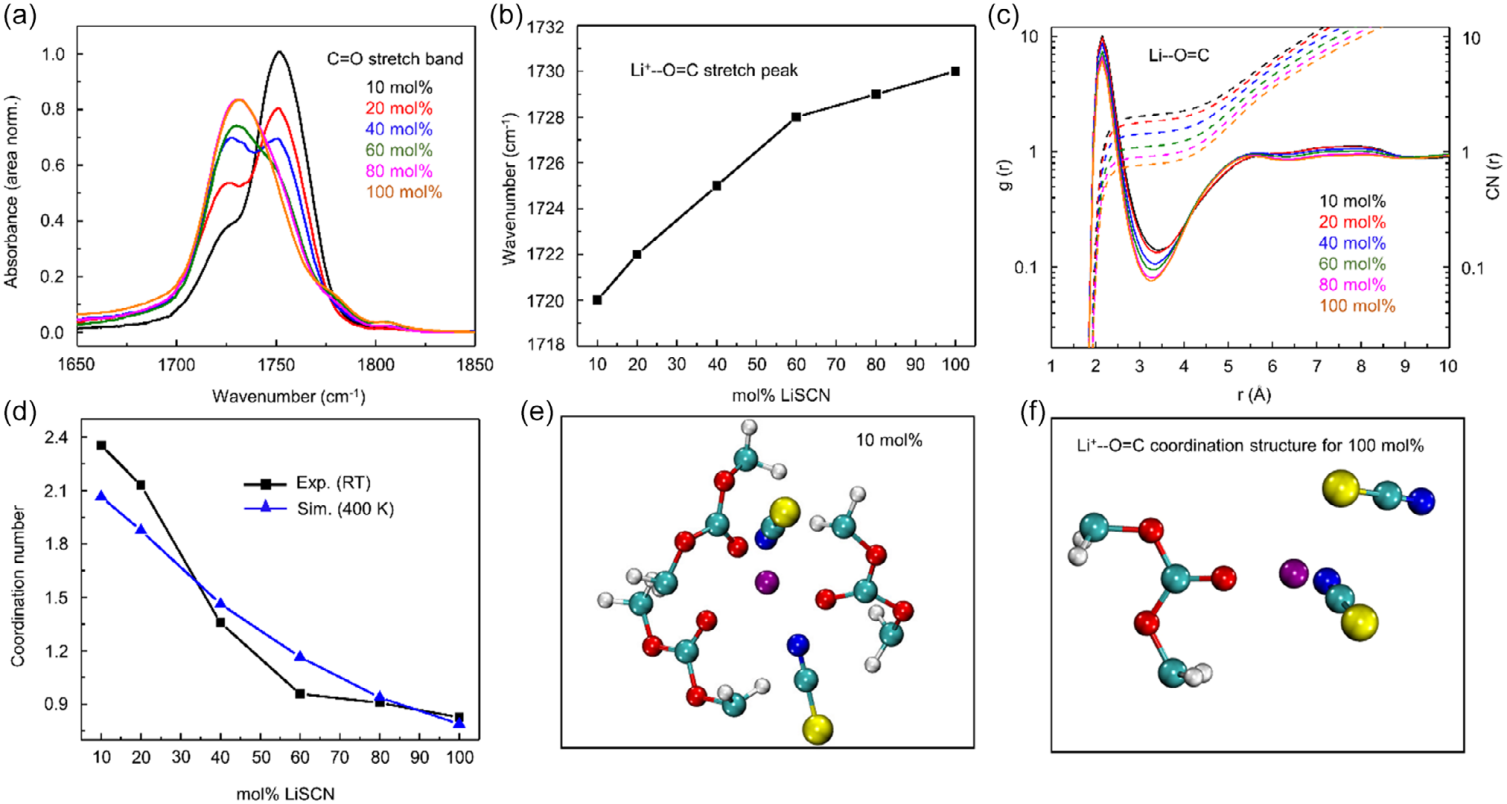
Li^+^–PEC solvation shell analyses. Experiment: Variation of a) area normalized FTIR absorption spectra of C=O stretch and b) Li^+^‐bound C=O stretch peak frequency with LiSCN mol%. Experiment and simulation: c) RDF and RCN between Li–O=C pair around the Li^+^ ion at different LiSCN mol% at 400 K. d) Coordination number of C=O in the first solvation shell of Li^+^ at different LiSCN concentrations determined from experiment at room temperature (black symbol) and simulations at 400 K (blue symbol). MD snapshot of Li–O=C coordination structure in first solvation shell of Li^+^ for e) 10 mol% and f) 100 mol% LiSCN.

The IR spectra (Figure S9a, Supporting Information) consist of two well‐separated bands: in 1600–1900 and 1950–2150 cm^−1^ regions, ascribed to C=O and C≡N stretch from PEC and SCN^−^, respectively, allow us to monitor the salt concentration‐dependent changes of the PEC (C=O)–Li^+^ (polymer backbone) and Li^+^–SCN^−^ (ion cluster) structure around Li^+^ ion. The IR spectra of pure PEC shown in the inset indicate that PEC has no overlapping IR absorption band in the SCN^−^ stretch region. The absence of a water IR band except at very high salt concentrations (80 mol% and above) in the 3200–3600 cm^−1^ range confirms negligible water contamination (Figure S9b, Supporting Information). As shown in **Figure** **3**a, area normalized C=O stretch IR spectra of PEC in LiSCN‐PEC SPE films consist of two peaks at ≈1724 and ≈1752 cm^−1^, described by two Voigt functions (Figure S10, Supporting Information), representing the Li^+^‐bound and free carbonyl, respectively. With increasing salt concentration, a 10 cm^−1^ blue shift (from 1720 to 1730 cm^−1^) observed for the Li^+^‐bound carbonyl peak (Figure 3b) manifests the gradual weakening of Li^+^‐carbonate interaction. This weakening of Li^+^–O=C interaction results in faster PEC segmental motion, a lower glass transition temperature, and a decrease in $E_{\text{BE}}^{{\text{Li}}^{+}}$ with increasing LiSCN concentration. Figure 3c represents the radial distribution function (RDF, *g*(r) and radial coordination number (RCN, CN(r)) of carbonyl oxygen atoms of PEC around the Li^+^ ion. The first maxima of RDF and the RCN within the first solvation shell decrease with increasing LiSCN mol%, indicating diminished Li^+^–PEC interaction with increasing salt concentration.

The weakening of the Li–polymer interaction, as evidenced by the blue shift in the C=O stretch frequency from IR experiments and the simulated Li‐polymer binding energy, competes with the slowdown of the polymer segmental relaxation timescale, influencing the ion transport mechanism between 80 and 100 mol% LiSCN. This could explain the temperature‐induced variation in ionic conductivity observed in Figure 1b and Table S1, Supporting Information: The values of *σ*_dc_ at 298 and 303 K are highest for 80 mol% LiSCN, but at higher temperatures (308, 313, 318, and 323 K), 100 mol% LiSCN shows the highest conductivity. This suggests a temperature‐induced transition in the transport mechanism from 80 to 100 mol% LiSCN. From Table S3, Supporting Information, we observe that at 298 K, the polymer segmental relaxation time for 100 mol% is ≈1.4 times slower than for 80 mol%, which reduces the expected ionic conductivity enhancement from weaker Li–polymer interaction, whereas at higher temperatures (≥308 K), the segmental motion timescales remain nearly identical for both 80 and 100 mol%, making the reduced Li–polymer interaction the primary factor governing the variation in ionic conductivity between 80 and 100 mol% and thus increased.

Figure S11, Supporting Information, shows a gradual increase and decrease of relative peak areas for Li^+^‐bound and free carbonyl stretch. As expected, the Li^+^‐bound carbonyl population grows with increasing salt concentration. The simulated relative population of free carbonyl and Li‐bound C=O follow similar variation. Figure 3d shows the carbonate coordination number (CN) in the first solvation shell of Li^+^ (Note S10, Supporting Information), which indicates nearly 2–3 carbonate units are coordinated with Li^+^ at 10 mol%, and CN gradually decreases to less than 1 as the salt content increases to 100 mol%. The carbonyl oxygen coordination number calculated from the MD simulation corroborates with experimental results (Figure 3d). The MD snapshots, as shown in Figure 3e,f, depict the Li^+^‐‐O=C coordination structure in the first solvation shell of Li^+^ for 10 and 100 mol% LiSCN, respectively. At 10 mol%, $f_{{\text{Li}}^{+}}$ (Table 1) indicates 98% of Li^+^ ions are coordinated to O=C, while this decreases around 60% at 100 mol% LiSCN. Therefore, at 100 mol%, around half of the total Li^+^ ions are surrounded only by SCN^−^. Consequently, we further examined Li^+^–SCN^−^ interaction via SCN^−^ IR spectra and MD simulation.

### 2.5 Signature of Ion Species and Ion Network Formation

**Figure 4:**
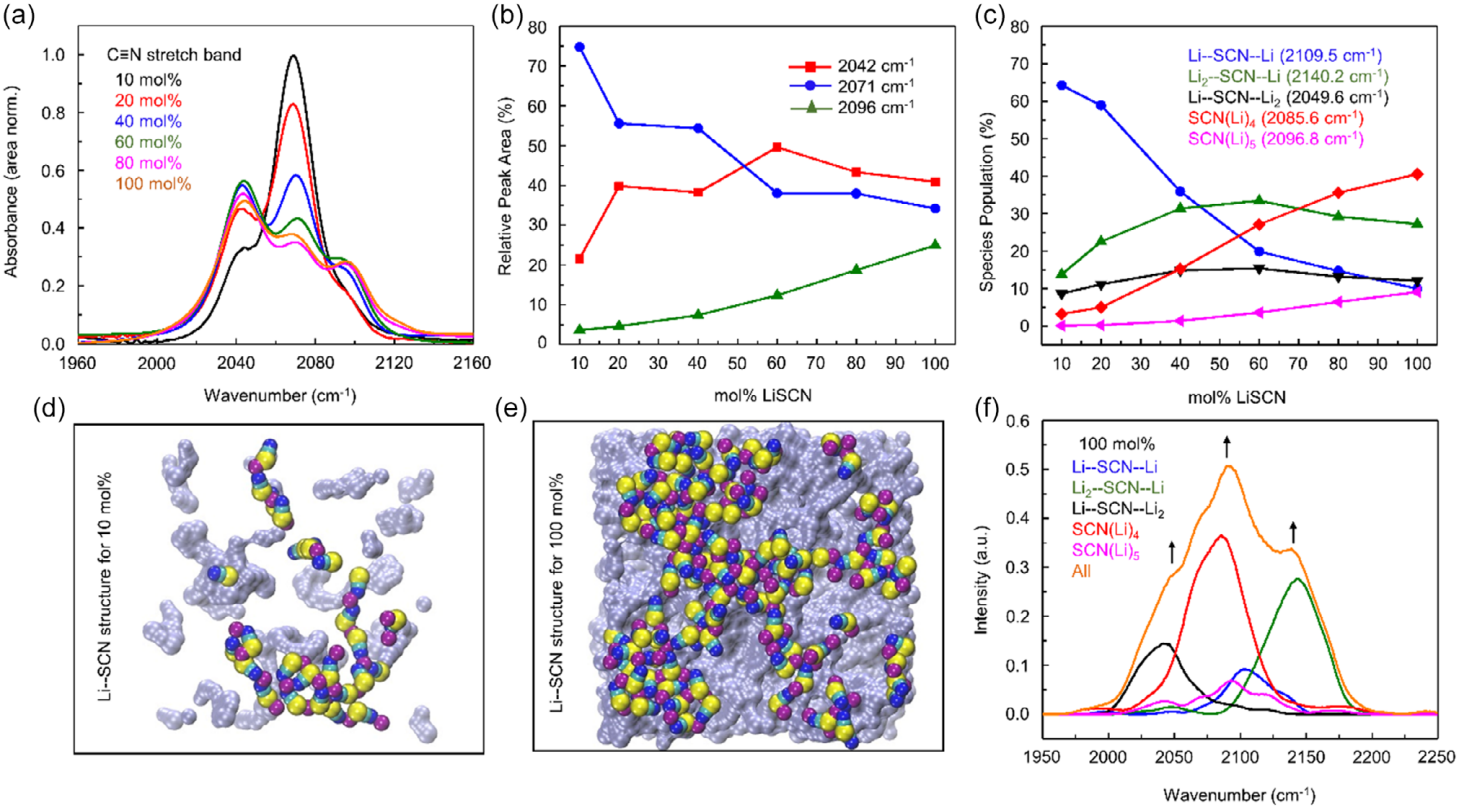
SCN^−^ centric solvation shell studies. Experiment: a) area normalized FTIR absorption spectra of SCN^−^ stretch vibration and b) LiSCN mol% dependent variation of peak area contribution for each peak of SCN^−^ stretch band. c) MD simulated population of [SCN(Li)_*n*_]^(*n*−1)+^ (*n* = 2–5) in first solvation shell at 400 K. Snapshot structures of Li–SCN aggregates and ion networks in d) 10 mol% and e) 100 mol% LiSCN in PEC. f) Simulated spectra of SCN^−^ vibration mode in [SCN(Li)_*n*_]^(*n*−1)+^ (*n* = 2–5) clusters for 100 mol% LiSCN. Arrows indicate spectral peaks corresponding to different clusters.

The C≡N stretch spectrum of the SCN^−^ in **Figure** **4**a consists of three dominating peaks ≈2042, 2071, and 2096 cm^−1^. Figure S12, Supporting Information, presents the deconvolution of C≡N stretch spectra using three Voigt functions. The absence of free SCN^−^ in LiSCN‐PEC SPE has been confirmed by inspecting the SCN^−^ band in liquid carbonate solvents (see Note S11, Supporting Information for detailed analysis). LiSCN concentration‐induced changes in the relative integrated areas for three peaks in LiSCN‐PEC SPEs, as shown in Figure 4b, indicate that with increasing LiSCN concentration, the 2071 cm^−1^ peak contribution diminishes, while the 2042 and 2096 cm^−1^ species increase significantly. The 2096 cm^−1^ species are present in small amounts at all concentrations, but their population increases with salt concentration.

The FTIR spectra of SCN^−^ ions in LiSCN‐PEC SPEs can be analyzed using the solvation structure around Li^+^ and SCN^−^ ions from MD simulation. Based on the first minima cutoff of the RDFs between the Li–N and Li–S pairs at different LiSCN mol% (Figure S15, Supporting Information), the solvation structures around Li^+^ and SCN^−^ are calculated. SCN^−^, being a bidentate ligand, can interact with Li^+^ either by S or N sites. The intensity at the first maxima gradually decreases with an increase in salt concentration, whereas CN(r) of N or S around the Li^+^ increases from ≈1.0 for 10 mol% to ≈2.0 for 100 mol%. This hints at compact ion‐cluster formation at low salt concentration, possibly ion‐network formation at higher LiSCN mol%. Figure 4c and Figure S16, Supporting Information, show that at 10 mol% LiSCN concentration, the first coordination shell of Li^+^ and SCN^−^ ions are mainly composed of SCN–Li–SCN and Li–SCN–Li motifs, respectively, indicating the formation of chain‐like ionic aggregates at low salt concentrations, as evidenced by the snapshot in Figure 4d. With increasing salt concentration, the population of the chain‐like motifs decreases, while (SCN)_2_ > Li < (SCN)_2_ and (Li)_2_ > SCN < (Li)_2_ motifs become the predominant species. This indicates the formation of a percolated ion network at high salt concentration, as seen in Figure 4e. Such a structural transition from small ionic aggregates to 3D extended ion networks was first established in aqueous electrolyte solutions of KSCN.^[10b]^ The SCN‐vibrational spectra project complex population switching among the corresponding ion species SCN(Li)_*n*_ (*n* > 1) into only three peaks due to insufficient spectral resolution. To extract the detailed structures, we simulated the vibrational spectra of the SCN^−^ stretch band (detailed protocols in Note S12, Supporting Information). The computed average frequencies for each motif are mentioned along with the legends in Figure 4c.

Figure S17, Supporting Information, represents the simulated spectra of SCN^−^ ions at different salt concentrations and corresponding deconvolution, respectively. For 10 mol%, the simulated spectrum appears as a single peak at ≈2110.0 cm^−1^ accompanied by two weak shoulders at ≈2050.0 and 2140.0 cm^−1^, closely resembling the experimental FTIR. With increasing salt concentration, the intensity of this central peak diminishes while the shoulder peaks become more prominent, as observed in experiments. To understand this spectral evolution with salt concentrations, we plotted the simulated C≡N stretch spectra for the 100 mol% LiSCN arising from different SCN(Li)_*n*_ motifs along with the sum of all in Figure 4f. As shown in Figure 4c, the average C≡N stretch of Li–SCN–Li motif appears at ≈2109.5 cm^−1^. Binding an extra Li^+^ ion at the sulfur or nitrogen end to this motif results in the formation of Li_2_ > SCN–Li and Li–SCN < Li_2_ complexes with average C≡N frequencies blue shifted to ≈2140.02 cm^−1^ and red shifted to 2049.6 cm^−1^, respectively. A similar observation was reported on vibrational solvatochromism of SCN^−^ in water where the vicinity of a water proton to the sulfur led to a blue shift of the C≡N stretch frequency, while interaction with nitrogen caused a red shift.^[^ ^13^ ^]^ Therefore, binding another Li^+^ at sulfur of Li–SCN < Li_2_ cluster will blue shift the C≡N stretch to ≈2085.6 cm^−1^ for the Li_2_ > SCN < Li_2_ cluster. The spectral response (≈2096.8 cm^−1^) of the SCN(Li)_5_ clusters indicates its structural similarity with the Li_2_ > SCN < Li_2_ motifs.

The concentration‐dependent evolution of SCN^−^ stretch band is a key signature of ion network formation in LiSCN‐PEC SPEs. The SCN^−^ stretch band reflects the structural transition from chain‐like ion‐cluster, Li–SCN–Li, to ion network structures, Li_2_ > SCN < Li_2_. In representative snapshots of the MD trajectory (Figure 4d,e), a clear transition from ion clusters at low LiSCN concentrations to percolating ion networks at higher concentrations is visible. As the concentration increases, the population of Li–SCN–Li motifs diminishes, and other motifs start to grow. The building block of the ion network, Li_2_ > SCN < Li_2_, becomes the dominant species. This population switching is the key factor behind the variation of relative peak intensities and the observed evolution of FTIR spectra can be understood from the concentration‐dependent evolution of different SCN(Li)_*n*_ motifs. The peak around 2100 cm^−1^ in the simulated spectrum (Figure 4f) is mainly contributed by Li_2_ > SCN < Li_2_ at higher concentrations and by Li–SCN–Li at lower concentrations, with a minor contribution from SCN(Li)_5_. The low‐frequency peak around 2050 cm^−1^ is primarily contributed by Li–SCN < Li_2_ at low concentrations, while significant contributions from Li_2_ > SCN < Li_2_ are added at high concentrations. The high‐frequency one around 2150 cm^−1^ can be assigned to Li_2_ > SCN–Li motifs. Thus, the experimentally observed center peak at 2071 cm^−1^ comes from a combination of Li–SCN–Li, Li_2_ > SCN < Li_2_, and SCN(Li)_5_. The band at 2042 cm^−1^ originates from a combination of Li–SCN < Li_2_ and Li_2_ > SCN < Li_2_, while the band at 2096 cm^−1^ is from Li_2_–SCN–Li motifs. Our previous studies on highly concentrated aqueous electrolytes showed these unresolved solvation species at 2042 cm^−1^ in the frequency domain could be resolved in the time domain with different vibrational lifetimes.^[^ ^14^ ^]^ The vibrational lifetime from IR pump‐probe experiments supports the assignment of 2042 cm^−1^ peak to Li_2_ > SCN < Li_2_ and Li–SCN < Li_2_ species (Note S13, Supporting Information).

## 3 Conclusion

We developed concentrated PEC‐based SPEs with LiSCN salt, which provides higher ionic conductivity compared to other Li salts with bulky anions like LiTFSI and LiFSI. By leveraging spectroscopic analysis and MD simulations, we offer an in‐depth molecular understanding of the mechanisms driving enhanced ion conductivity with increasing LiSCN concentration. With increasing LiSCN concentration, the reduced Li^+^–PEC interaction promotes faster polymer segmental motion and more efficient Li^+^ ion transfer along the polymer backbone. Moreover, the strong tendency of SCN^−^ to form ion aggregates partitions more Li^+^ ions into the ion network channels, thereby enhancing charge transport through this network. At higher LiSCN concentrations, a percolated ion network channel is formed, facilitating faster Li^+^ diffusion. This conclusion, previously suggested through MD simulations alone, is now experimentally validated using vibrational spectroscopy. These insights suggest that the inherent limitations of SPEs in terms of ionic conductivity can be mitigated by creating additional fast Li^+^ diffusion channels. This can be achieved not only through high concentrations of LiSCN but also potentially by incorporating multiple salts or inorganic additives into the electrolyte matrix.

## 4 Experimental Section

The materials and methods used in this study have been described in detail in the Supporting Information. In brief, purchased polyethylene carbonate (PEC, molecular weight 169 000, Empower Materials) was further purified by dissolving in acetonitrile (ACN, Sigma–Aldrich, purity ≥99.5%) followed by precipitation in excess methanol (Daejung Chemicals, purity ≥99.5%). The precipitated PEC was vacuum dried at 60 °C for 24 h and the purified PEC was stored in a glove box for use. The hydrated LiSCN (LiSCN·*x*H_2_O, Sigma–Aldrich) was dried in a vacuum oven by gradually increasing the oven temperature from room temperature to 110 °C throughout a week. The anhydrous LiSCN was stored in the glovebox for use. In the present study, the concentration of LiSCN salt was set to be in the range from 10 to 100 mol% (*x* mol%, $x=\frac{\left[\text{LiSCN}\right]}{\left[\text{EC unit}\right]}\times 100$). For instance, 10 mol% LiSCN means that Li/carbonate molar ratio equals 1/10. To prepare the SPE films, anhydrous LiSCN and PEC were dissolved in a binary solvent mixture of acetonitrile (ACN): dichloromethane (DCM) (anhydrous, Sigma–Aldrich). Spin coating and solution casting method were applied for FTIR and dielectric relaxation spectroscopy, respectively. Transmission linear FTIR (PerkinElmer Frontier FT‐MIR Spectrometer) and femtosecond Mid‐IR pump‐probe spectroscopy and electrochemical impedance spectroscopy techniques (HIOKI LCR meter (model: IM3536) and probe L2000 in the frequency regime 4 Hz to 8 MHz with 100 mV amplitude) were employed in this study for exploring ion transport mechanism in LiSCN‐PEC SPEs. Detailed information about the instrumentation is available in Note S1, Supporting Information.^[^ ^15^ ^]^

## Conflict of Interest

The authors declare no conflict of interest.

## Author Contributions

**Seoeun Shin**: data curation (supporting); formal analysis (supporting); methodology (supporting); writing—review and editing (supporting). **So Yeon Chun**: data curation (supporting); methodology (supporting); writing—review and editing (supporting). **Joong Won Shim:** data curation (supporting); formal analysis (supporting); methodology (supporting); writing—review and editing (supporting). **Kyung‐Koo Lee**: conceptualization (supporting); investigation (supporting); methodology (supporting); resources (equal); supervision (equal); validation (equal); visualization (equal); writing—review and editing (supporting). **Minhaeng Cho**: conceptualization (equal); funding acquisition (lead); investigation (equal); methodology (equal); resources (lead); supervision (equal); validation (equal); visualization (equal); writing—review and editing (equal). **Kyungwon Kwak**: conceptualization (equal); funding acquisition (equal); investigation (equal); methodology (equal); project administration (equal); supervision (lead); validation (equal); writing—review and editing (equal). **Kajal Kumbhakar** and **Sourav Palchowdhury** contributed equally to this work.

## Supporting information

Supplementary Material

## Data Availability

The data that support the findings of this study are available in the supplementary material of this article.
