# Supplementary material for: Enhanced Charge Transport through Ion Networks in Highly Concentrated LiSCN‐Polyethylene Carbonate Solid Polymer Electrolytes
Source: Small Sci. 2025 Jan 25;5(6):2400653. doi: 10.1002/smsc.202400653 (PMC12168596; doi:10.1002/smsc.202400653)
Supplement: Supplementary file 1 — Supplementary Material [file SMSC-5-2400653-s001.pdf]

SUPPORTING INFORMATION

Enhanced Charge Transport Through Ion Networks in Highly Concentrated LiSCN-polyethylene Carbonate Solid Polymer Electrolytes

Kajal Kumbhakar,[a],† Sourav Palchowdhury,[a],† Thuy Duong Pham,[b] Seoeun Shin,[a] So Yeon Chun,[a] Joong Won Shim,[a],[c] Kyung-Koo Lee,[d],* Minhaeng Cho,[a],[c],* Kyungwon Kwak[a],[c],*

[a] Dr. K. Kumbhakar, Dr. S. Palchowdhury, S. Shin, Dr. S. Y. Chun, J. W. Shim, Prof. M. Cho, Prof. K. Kwak
Center for Molecular Spectroscopy and Dynamics
Institute for Basic Science (IBS)
Seoul 02841, Republic of Korea
E-mail: mcho@korea.ac.kr, kkwak@korea.ac.kr

[b] Dr. T. D. Pham
Faculty of Biotechnology
Chemistry and Environmental Engineering
Phenikaa University, Hanoi 10000, Vietnam

[a], [c] J. W. Shim, Prof. M. Cho, Prof. K. Kwak
Department of Chemistry
Korea University
Seoul 02841, Republic of Korea
E-mail: mcho@korea.ac.kr, kkwak@korea.ac.kr

[d] Prof. K. -K. Lee
Department of Chemistry
Kunsan National University
Gunsan, Jeonbuk 54150, Republic of Korea
E-mail: kklee@kunsan.ac.kr

†K. Kumbhakar and S. Palchowdhury contributed equally to this work

Table of Contents

Supporting Note 1. Experimental Method........S2
Supporting Note 2. Analyses of DC ionic conductivity and polymer segmental motion........S4
Supporting Note 3. MD simulation methodology details........S5
Supporting Note 4. Translational charactristics analses........S6
Supporting Note 5. Ion translational mobilities and simulated binding energies in LiFSI-PEC and LiTFSI-PEC SPEs........S6
Supporting Note 6. Determination of activation energies for ionic conductivity........S7
Supporting Note 7. Calculation of molar ionic conductivity........S7
Supporting Note 8. MSD of a monomer of PEC........S7
Supporting Note 9. MD simulated relaxation kinetics around O–C–C–O dihedral........S7
Supporting Note 10. Carbonate coordination number surrounding Li-ion........S7
Supporting Note 11. C≡N stretching band in LiSCN-PC (or DMC) solutions........S8
Supporting Note 12. Simulation of vibrational spectra of SCN^-........S8

Supporting Note 3. Pump-probe infrared measurements……S8
Table S1. DC ionic conductivity and activation energies in x mol% LiSCN-PEC SPEs……S9
Table S2. Decomposition of average binding energy at 400 K for LiFSI-PEC and LiTFSI-PEC SPEs……S9
Table S3. Polymer segmnetal relaxation times in LiSCN-PEC SPE films……S9
Table S4. The values of thickness, area, volume and molar concentration for x mol% LiSCN-PEC SPE films……S10
Table S5. Molar ionic conductivity of x mol% LiSCN-PEC SPE films at various temperature……S10
Table S6. Diffusion coefficients (D) of carbonyl oxygens and Rouse model fit parameters for MSDs of EC monomer at 400……S10
Table S7. The fit parameters corresponding to O–C–C–O dihedral relaxation at 400 K……S10
Table S8. The relative integrated area for Li^+-bound and free C=O stretch band and carbonyl coordination number……S10
Table S9. Excited state vibrational lifetime of C≡N stretch mode in different SCN^- species present in LiSCN-PEC SPEs……S11
Supporting Scheme 1: Chemical structures of polyethylene carbonate, lithium thiocyanate and monomer EC……S11
Figure S1. Impact of residual solvent on ionic conductivity of LiSCN-PEC SPEs……S11
Figure S2. Impedance spectra in LiSCN-PEC SPEs in Nyquist presentation……S12
Figure S3. MSDs of Li^+, SCN^-, Nernst-Einstein and collective ionic conductivities as a function of LiSCN concentrations at 400 K……S12
Figure S4. Comparison of MD simulated collective ionic conductivity between LiSCN-PEC, LiFSI-PEC, and LiTFSI-PEC SPEs, MSD and translational self-diffusion coefficient of cation and anions in LiFSI-PEC and LiTFSI-PEC SPEs at 400 K……S13
Figure S5. Real ($\varepsilon'(f)$) and imaginary ($\varepsilon''(f)$) component of complex dielectric spectra in LiSCN-PEC SPEs……S13
Figure S6. τ_s, T_g and DSC thermogram of LiSCN-PEC SPE films and pure PEC……S14
Figure S7. Arrhenius plot of experimental σ_{dc}……S14
Figure S8. MSDs of EC monomer unit of PEC chain and O–C–C–O dihedral relaxation at 400 K……S14
Figure S9. FTIR absorption spectra of LiSCN-PEC SPE films with different salt concentrations and IR absorption spectrum around O-H stretching mode in those SPEs……S15
Figure S10. Deconvolution of FTIR absorption spectra of C=O stretch in LiSCN-PEC SPE films……S15
Figure S11. Relative peak area of free C=O and Li^+-bound C=O from experiment and relative population of free C=O and Li^+-bound C=O from simulation at different LiSCN mol%……S16
Figure S12. Deconvolution of FTIR absorption spectra of CN stretch in LiSCN-PEC SPE films……S16
Figure S13. FTIR absorption spectra of SCN^- stretch in LiSCN-PC at different salt concentration and their deconvolution……S16
Figure S14. FTIR spectral analyses in LiSCN-DMC mixture……S17
Figure S15. The radial distribution function and radial coordination number between the Li--N pair and Li--S pair at 400 K……S17
Figure S16. MD simulated population of $[Li(SCN)_n]^{(n-1)-}$ (n=2-5) in the first solvation shell at 400 K……S18
Figure S17. Simulated spectra of SCN^- vibration mode in $[SCN(Li)_n]^{(n-1)+}$ (n= 2-5) clusters for at different LiSCN mol%……S18
Figure S18. Isotropic IR pump-probe analyses of C≡N stretch mode of SCN^- in LiSCN-PEC with different salt concentration……S19
Supporting References……S19

Supporting Note 1. Experimental Method

FTIR spectroscopy. FTIR spectra were collected with a PerkinElmer Frontier FT-MIR Spectrometer in the transmission mode. For each FTIR experiment, four scans were performed at a resolution of 2 cm^{-1}. All FTIR measurements were performed at room temperature under nitrogen gas purging conditions.

Electrochemical impedance spectroscopy (EIS). Impedance spectra of the solid polymer electrolyte films were recorded using the HIOKI LCR meter (model: IM3536) and probe L2000 in the frequency regime 4 Hz to 8 MHz with 100 mV amplitude. A lab-customized temperature controller has been used for temperature-dependent EIS measurements.

Femtosecond Mid-IR pump-probe spectroscopy. Ti:Sapphire oscillator (Maitai, Spectra Physics) and a regenerative amplifier (SolsticeAce, Spectra Physics) produce 800 nm laser pulses with a repetition frequency of 1 kHz. BBO crystal-based optical parametric amplifier (OPA) system generates two near-IR pulses with wavelengths of approximately 1.3 and 2.0 μm. Mid-IR (MIR) pulses with a center wavenumber (2050 or 1725 cm^{-1}) are generated via a difference-frequency-generation process induced by an $AgGaS_2$ crystal.

The generated MIR pulse has an approximately 35 fs pulse duration time and about 29 J energy. Note that the Ge plate was used to remove the two near IR pulses (which are generated by OPA, signal, and idler pulses). Furthermore, the thicknesses of Ge plates and CaF2 windows are controlled to compensate for the chirp between pump and probe pulses. We used a ZnSe beam splitter (9:1) to produce both pump and probe pulses to carry out polarization-selective IR-PP measurements. The repetition rate of the pump pulse was adjusted to 500 Hz using an electronically synchronized optical chopper. The two MIR pulses were illuminated on samples through different beam paths and the time delay between pulses was controlled via retroreflectors mounted on motorized delay stages. With wire grid polarizers, the polarization angle between probe and pump pulses was set to be 45°, making the probe pulse consist of identical contributions of parallel (0°) and perpendicular (90°) components with respect to pump polarization. After the interactions of the pump and probe pulse with the sample, the probe pulse was divided into parallel and perpendicular polarization components with respect to the pump polarization using a ZnSe beam splitter (5:5) and wire grid polarizer. The probe pulse can monitor the pump-induced changes in vibrational states in sample molecules, which is measured by a monochromator (Horiba, iHR320) and 128(64*2)-element array MCT detector. The parallel polarization component of the probe beam was detected by the bottom array of the dual array MCT detector. The perpendicular polarization component of the probe beam was detected by the upper array. We measured both the 0-1 transition (ground-state bleach and stimulated emission contributions to the IR PP) and the 1-2 transition (excited-state absorption contribution to the IR-PP) of the C≡N/carbonyl stretch vibration. With polarization-selective IR-PP signals (i.e., parallel ($\Delta\alpha\parallel$) and perpendicular signal ($\Delta\alpha\perp$)), one can obtain the vibrational population relaxation of the vibrational probe. The equations of the isotropic (vibrational relaxation) signal are given by

$$P(t)=\Delta\alpha_{\parallel}+2\Delta\alpha_{\perp} \quad \text{(S1)}$$

where $P(t)$ is the vibrational population relaxation.

Differential scanning calorimetry (DSC). Differential scanning calorimetry scans were recorded with DSC 250 (TA instrument) to measure glass transition temperature (T_g) of the SPE films. The temperature was scanned with a rate of 10°C/min. T_g has been determined from the second heating scan of the DSC thermogram.

Materials and sample preparation. Purchased polyethylene carbonate (PEC, molecular weight 169000, Empower Materials) was further purified by dissolving PEC in acetonitrile (ACN, Sigma Aldrich, purity ≥99.5%) under continuous stirring for 4 hours at 55°C. Then, this solution was added dropwise in excess methanol (Daejung Chemicals, purity ≥99.5%) to precipitate out the PEC. The precipitated PEC was vacuum-dried at 60°C for 24 hr. The dried PEC was stored in a glove box for use. LiSCN is highly sensitive to moisture; in the presence of moisture, LiSCN may degrade to yellow S with rapidly increasing temperature under vacuum drying conditions. To prevent such LiSCN degradation, hydrated LiSCN ($LiSCN.xH_2O$, Sigma Aldrich) dried in a vacuum oven by gradually increasing the oven temperature from room temperature to 110°C throughout a week. The anhydrous LiSCN was stored in the glovebox for use. In the present study, the concentration of LiSCN salt was set to be in the range from 10 to 100 mol% (x mol%, $x = \frac{[\mathrm{LiSCN}]}{[\mathrm{EC\ unit}]}\mathrm{x}100$). For instance, 10 mol% LiSCN means that Li/carbonate molar ratio equals 1/10.

Sample preparation for transmission FT-IR experiment. Anhydrous LiSCN and PEC were dissolved in a binary solvent mixture of acetonitrile (ACN): dichloromethane (DCM) (anhydrous, Sigma Aldrich). The SPE films were prepared on a CaF_2 IR window by spin coating method at 8000 rpm for 300 s. The spin coating results in an SPE thickness of ~ 10-20 µm with negligible residual solvent. A second CaF_2 IR window is used to make a sandwich IR cell like CaF_2‖SPE‖CaF_2, and the edge of this sandwich cell is sealed with parafilm to avoid contamination with moisture during the FTIR experiment. Subsequently, the FTIR cell was assembled to record FTIR spectra. The sample preparation and cell assembly process were carried out inside the glovebox to avoid moisture absorption by the LiSCN salt.

Sample preparation for electrochemical impedance spectroscopy (EIS) measurement. Binary solvent mixture of ACN:DCM was added to dissolve LiSCN and PEC. 0.2 mL solution of LiSCN-PEC in ACN:DCM mixture was casted on stainless steel (SS) circular spacer disk (diameter 16 mm, thickness ~ 0.5 mm) and left for 24 hours at room temperature for solvent evaporation. High-temperature vacuum drying was avoided due to the highly hygroscopic nature of LiSCN, as even trace amounts of moisture can cause degradation under such conditions due to its tendency to readily absorb water. To maintain the stability and performance of the SPEs, we opted for

room temperature drying methods and minimized moisture exposure. Due to the use of volatile solvent DCM as a major volume ratio, the dried SPEs contain very little residual solvent, around 5-15 wt%. Due to the use of volatile solvent DCM as a major volume ratio, the dried SPEs contain very little residual solvent, around 5-15 wt%. The residual solvent content was determined by weight measurement of the SPEs before and after the film dry. Then, the coin cell was prepared with the following configuration: SS spacer‖ LiSCN-PEC film ‖SS spacer. The thickness of the SPE films is ~ 90-130 µm with an area of 2.01 cm^2. Temperature-dependent impedance measurements were performed from 298 K to 323 K. Before recording EIS data at a particular temperature, sufficient time (15 min after reaching the set temperature) was allowed to achieve thermal equilibration.

Note that the solution casting method for making SPEs cannot remove residual solvent completely. A two-order decrease of ionic conductivity in PAN:LiTFSI SPEs was reported when residual solvent content decreased from 23 to 5 mol%.[1] A certain amount of residual solvent can increase the ionic conductivity, cycling stability, and capacity and thus can benefit their application.[2] In our present LiSCN-PEC SPEs, we showed a 5-50 times increase of ionic conductivity at 298 K when residual solvent increased from ~ 5-15 wt% to more than 10-15 wt% (Figure S1). Generally, residual solvent in SPEs increases with the addition of Li salts due to ion-dipole interactions. As a result, SPEs with higher Li salt concentrations tend to exhibit higher ionic conductivity. The presence of residual solvent complicates the determination of whether the enhanced conductivity is due to a fundamentally different ion transport mechanism or simply a result of the residual solvent. The reported enhancement in ionic conductivity with increasing salt concentration in LiTFSI-PEC, LiFSI-PEC, and $LiClO_4$-PEC [3] where the residual solvent is negligible (due to high-temperature vacuum film drying), indicates that ionic conductivity indeed increases with salt concentration. Nevertheless, the LiSCN concentration–dependent ionic conductivity (Fig. 1b) obtained from MD simulations—where no residual solvent is considered—still qualitatively match the experimental findings (Table S1), which do include residual solvent. Hence, we conclude that residual solvent does not significantly hinder our understanding of the LiSCN concentration–dependent ion transport mechanism in these SPEs

The standard film preparation procedure[3c] was applied for LiTFSI-PEC and LiFSI-PEC SPE films for EIS studies.

Supporting Note 2. Analyses of DC Ionic Conductivity and Polymer Segmental Motion. Frequency-dependent complex impedance spectra ($Z^*(\omega)$, radial frequency $\omega = 2\pi f$, f is the frequency in Hz) collected from impedance analyzer. Representative $Z^*(\omega)$ in Nyquist's presentation is shown in Figure S2a. The compex impedance plots for the SPEs were fitted to an equivalent circuit model shown in the Figure S2b, which consist of bulk resistance (R_b) and constant phase elecment to the bulk capacitance (CPE_b) for the semi-circle region and another CPE_i for the slanted line (spike) in the low-frequency region referred to interfacial polarization due to the blocking effect of the electrodes. The ionic conductivity, shown in parentheses in Table S1, calculated from the fitted parameter R_b following the equation:

$$\sigma_{dc} = \frac{1}{R_b} \text{x} \frac{d}{A} \quad \text{(S2)}$$

Here d and A are the thickness and area of solid polymer electrolyte films. These are equivalent to the distance between two electrodes of the cell, d, and the electrode surface area A, respectively.

The $Z^*(\omega)$ data has been further transformed to frequency-dependent complex conductivity $\sigma^*(\omega)$ and dielectric constant ($\varepsilon^*(\omega)$). $Z^*(\omega)$, $\sigma^*(\omega)$ and $\varepsilon^*(\omega)$ is defined as:

$$Z^*(\omega) = Z'(\omega) + iZ''(\omega) \quad \text{(S3)}$$

where the real part, Z' is resistance, while the imaginary part, Z'', is known as reactance.

$$\sigma^*(\omega) = \sigma'(\omega) + i\sigma''(\omega) = \frac{1}{Z^*(\omega)} \text{x} \frac{d}{A} \quad \text{(S4)}$$

$$\sigma'(\omega) = \frac{Z'(\omega)}{\left(Z'^2+Z''^2\right)} \text{x} \frac{d}{A},\ \sigma''(\omega) = \frac{-Z''(\omega)}{\left(Z'^2+Z''^2\right)} \text{x} \frac{d}{A} \quad \text{(S5)}$$

$\sigma'(\omega)$ and $\sigma''(\omega)$ are, respectively, the real and imaginary components of $\sigma^*(\omega)$. In the real part of complex conductivity ($\sigma'(\omega)$), a frequency-independent plateau regime appears on the low-frequency side, and σ_{dc} is the extrapolation of the plateau to $\omega \to 0$. The extracted σ_{dc} in LiSCN-PEC SPEs with varying LiSCN mol% are summarized in Table S1.

$$\varepsilon^*(\omega) = \varepsilon'(\omega) - i\varepsilon''(\omega) = \frac{1}{i\omega Z^*(\omega) C_0} \quad \text{(S6)}$$

$$\varepsilon'(\omega) = \frac{-Z''(\omega)}{\omega C_0\left(Z'^2+Z''^2\right)} \text{ and } \varepsilon''(\omega) = \frac{Z'(\omega)}{\omega C_0\left(Z'^2+Z''^2\right)} \quad \text{(S7)}$$

Here ε' is the real part of $\varepsilon(\omega)$ known as dielectric permittivity (measures the energy stored), while the imaginary component ε'' is called dielectric loss (energy dissipated in the sample). $C_0 = \varepsilon_0 \frac{A}{d}$, is the capacitance of the empty cell. ε_0 (8.854 x 10^{-12} F/m) is the vacuum permittivity.

Figure S5 shows frequency-dependent real ($\varepsilon'(f)$) and imaginary components $\varepsilon''(f)$ of complex dielectric spectra ($\varepsilon^*(f)$) of LiSCN-PEC SPEs with varying LiSCN mol% at a representative temperature, 298 K. $\varepsilon'(f)$ spectra in Figure S5a reflect the typical frequency-dependent signature for ion-conducting sample: dipolar reorientation polarization contribution in the high-frequency regime and a sharp rise in the low-frequency regime. The sharp rise of $\varepsilon'(f)$ in the low-frequency window is the manifestation of the electrode polarization process due to charge accumulation at the electrode surface. In $\varepsilon''(f)$, because of ionic conductivity, the loss peak due to dipolar polarization relaxation is hidden, and $\varepsilon''(f)$ sharply rises (Figure S5c) with decreasing frequency according to the relation $\varepsilon''(f) = \frac{\sigma'(f)}{2\pi f \varepsilon_0}$.

To get the dipolar reorientation polarization relaxation information in the present SPEs, we excluded the electrode polarization contribution region during data fitting.

Dipolar polarization contribution due to dipoles in the polymer segment is observed in the high-frequency end[4]. In the ion-conducting SPEs, dipolar relaxation contribution in $\varepsilon^*(\omega)$ can be well described by the following empirical Havriliak-Negami function as (Figure S5b and d:[4-5]

$$\varepsilon^*(\omega) = \varepsilon_\infty + \frac{\Delta\varepsilon}{[1+(i\omega\tau_{HN})^\alpha]^\beta} + \frac{\sigma_{dc}}{i\varepsilon_0\omega} \quad \text{(S8)}$$

ε_∞ is the value of $\varepsilon'(f)$ at infinite frequency, $\Delta\varepsilon = \varepsilon_s - \varepsilon_\infty$ where ε_s (static dielectric constant) is the $\varepsilon'(f)$ at frequency near to zero. $\Delta\varepsilon$ is called dielectric relaxation strength of the dipolar relaxation process with characteristic relaxation time τ_{HN}. Note in polymeric system the dielectric relaxation curve is broad and asymmetry compared to Debye relaxation curve. α and β, respectively, accounts for the symmetric and asymmetric broadening of the complex dielectric function with $0 < \alpha, \alpha\beta \leq 1$. σ_{dc} is the DC conductivity. Note σ_{dc} obtained from the fitting using equation S8 can correctly describe the σ_{dc} predicted from the extrapolation of the frequency-independent plateau region of $\sigma'(\omega)$ to $\omega \to 0$. The segmental relaxation time (τ_s) is calculated from HN relaxation time and shape parameters (*α*, ***β***) using the following equation:

$$\tau_s = \tau_{HN} \frac{\left(\sin\frac{\alpha\beta\pi}{2+2\beta}\right)^{\frac{1}{\alpha}}}{\left(\sin\frac{\alpha\pi}{2+2\beta}\right)^{\frac{1}{\alpha}}} \quad \text{(S9)}$$

τ_s in LiSCN-PEC SPEs with varying LiSCN mol% are summarized in Table S3.

Supporting Note 3. Molecular Dynamic (MD) Simulation Methodology and Simulation Details. The initial configurations of LiSCN-PEC SPEs were obtained using the EMC software,[6] which generates more realistic entangled polymer chains. Each PEC chain consisted of 250 monomer units, while four chains were packed in a cubic box. The SPEs comprised 99, 197, 395, 592, 792, and 990 LiSCN ion pairs, resulting in 10 mol% to 100 mol% salt concentrations. Classical molecular dynamics simulations were performed with the LAMMPS package[7] using a fixed-charge non-polarizable OPLS force field parameter set. We have adopted the force field parameters derived for dimethyl carbonate (DMC) to represent the monomer units of PEC chains where the linker methylene carbon adopts the site charge on methyl hydrogen of the DMC,[8] while the ion pair force field parameters were taken from the literature.[9] All the atomic partial charges were scaled by 0.80 as suggested to correctly quantify the structural and transport properties of Li^+ ions in carbonate-based electrolytes.[9b] These SPE electrolytes, being dynamically constipated systems, were attempted to simulate at 400 K temperature. The initial configurations were equilibrated under the NVT ensemble for 4 ns followed by density equilibration for 20 ns under the NPT ensemble at 1 atm pressure. The last configurations of these density equilibrated systems were then subjected to 200 ns MD run under NVT ensemble, keeping the box length fixed at the values averaged over the last 1 ns of NPT simulations. The equations of motion were integrated using the Verlet method with a 2 fs time step. Bonds connected to hydrogen atoms and angles flanked by two hydrogen atoms were constrained via the SHAKE algorithm.[10] A periodic boundary condition was applied to all three dimensions. Nose-Hoover thermostat and barostat[11] with 1 ps and 2 ps damping parameters were used to control system temperature and pressure. Long-range electrostatic interactions were calculated using the particle-particle-particle-mesh method with a k-space tolerance of 10^{-5}.[12] VMD software was used to watch the trajectories.[13] The structural and dynamic properties were calculated from the last 100 ns of the NVT trajectories. To understand the degree of coupling between the ionic mobility and segmental motion of the polymer backbone, we extended our simulated systems to an additional 200 ns NVT run, where the polymer backbone was kept frozen

by initializing the velocity and setting the computed force on the polymer atoms to zero at each time instant. To compare the simulated ionic conductivity of LiSCN-PEC SPEs with LiTFSI-PEC and LiFSI-PEC at the same salt mol%, we have also simulated LiTFSI-PEC and LiFSI-PEC SPEs under the same protocol while using the force field parameters for TFSI and FSI anions from the literature.[9b]

Supporting Note 4. Translational Self-diffusion Coefficient, Nernst-Einstein Conductivity, Collective Ionic Conductivity. The translational self-diffusion coefficient has been calculated from the slope of mean-squared displacement (MSD) of the ions using the Einstein expression:

$$D = \frac{\langle |r_i(t) - r_i(0)|^2 \rangle}{6t} \quad \text{(S10)}$$

where $r_i(t)$ and $r_i(0)$ are the position vectors of the ion i at time t and origin, respectively, and the slope is calculated from the diffusive regime of the MSD plot.

We computed Nernst-Einstein ionic conductivities (σ_{NE}) for the SPEs, considering independent ion motion, which is equivalent to conductivities obtained from NMR experiments.[14]

$$\sigma_{NE} = \frac{e^2(N_+ Z_+^2 D_+ + N_- Z_-^2 D_-)}{V k_B T} \quad \text{(S11)}$$

where z_+, z_-, N_+ and N_- are the valency of cation, anion and numbers of cation and anion, respectively.

We computed ionic conductivities accounting for correlated ion motions using the Einstein form of the equation[15]

$$\sigma_{col} = \frac{e^2}{6tVk_BT} \sum_{i,j}^{n} \langle (z_i \Delta R_i(t)).(z_j \Delta R_j(t)) \rangle \quad \text{(S12)}$$

where e, V, k_B, and T are the elementary charge, volume of the system, Boltzmann constant, and temperature, respectively. $\Delta R_i(t)$ is the displacement vector of particle i during time t, z_i is the valency of ion i with a sign, and the sum runs over for all self and distinct pairs of ions in the system, representing the MSD arising from the collective ion motions.

Supporting Note 5. Ion Translational Mobilities in Different SPEs. The translational diffusion coefficients of cations and anions computed from the slope of MSDs in the diffusive regime of ions for the LiFSI-PEC and LiTFSI-PEC SPEs as a function of salt mol% are shown in Figure S4(d). The corresponding MSDs are also shown in Figure S4(b) and 4(c) for LiFSI-PEC and LiTFSI-PEC, respectively. For both the SPEs, although there is an initial drop of translational diffusion coefficients of Li^+ ions from 10 mol% to 20 mol% salt concentration, it keeps on increasing at higher salt mol%. We should note that the translational diffusion coefficients of Li^+ ions of the simulated SPEs decrease in the order LiSCN-PEC>LiFSI-PEC>LiTFSI-PEC, which is in the reverse order of anion sizes. Both FSI and TFSI anions translate slower than the Li^+ ions in the SPEs, contrary to what was observed in LiSCN-PEC SPEs. Also, the trend of translational diffusion coefficients of anions shows a non-monotonic behavior with salt mol%. The anion translational diffusion coefficients in both of these SPEs become less correlated with that of Li^+ as the salt concentration increases. These observations indicate that Li^+ ions are the major charge carriers in these two SPEs. It is important to note that the enhancement of translational diffusion coefficients of ions with decrease in anion size is the determining factor for the variation in observed ionic conductivities among these three classes of SPEs.

Simulated Binding Energies in LiFSI-PEC and LiTFSI-PEC. The simulated binding energies for Li^+ in LiFSI-PEC and LiTFSI-PEC are tabulated in Table S2. We can see that the total BE of Li^+ in LiSCN-PEC SPEs at a given mol% of salt is larger than the other two SPEs owing to more considerable contribution from ionic terms due to stronger interaction between Li^+ and smaller SCN^- than with the other two more polarizable FSI and TFSI anions. The energy decomposition analyses clearly show that the Li^+--PEC interaction at a given salt mol% increases in the order LiSCN-PEC<LiFSI-PEC<LiTFSI-PEC. In other words, the larger the ionic interaction strength to the total binding energy of Li^+, the more Li^+ ions are fetched from the polymer vicinity to the ionic domain, leading to a decrease in Li^+--PEC interaction strength. We believe that the decrease in Li^+--PEC interaction strength in LiSCN-PEC as compared to the other two SPEs is the underlying energetic contribution to the enhanced Li^+-diffusivity and hence ionic conductivity in the former and also governs the overall trend.

Supporting Note 6. Determination of Activation Energies. We determined activation energies of conductivity ($E_a^{\sigma_{dc}}$) from the Arrhenius-type fitting following the equation: $\sigma_{dc} = \sigma_{dc}^{\infty} exp\,(-\frac{E_a^{\sigma_{dc}}}{RT})$, as shown in Figure S7a, where σ_{dc}^{∞} is the pre-exponential factor. The activation energies are summarized in Table S1, where $E_a^{\sigma_{dc}}$ decreases with increasing LiSCN concentration up to 80 mol%.

Supporting Note 7. Calculation of Molar Ionic Conductivity. Apparent molar ionic conductivity is defined by $\Lambda = \frac{\sigma_{dc}}{c}$. Here c is the molar concentration of LiSCN dissolved in polymer PEC, defined with mol cm^{-3} unit. From the volume of the LiSCN-PEC film and LiSCN mole content in that film, we calculated c. Considering the cylindrical shape, the volume (*V*) of the film can be estimated by surface area of the SS spacer (*A*) x thickness of the film (*d*). The values of *d*, *A*, *V*, and n_{tot} are summarized in Table S4. The molar ionic conductivities of LiSCN-PEC films are provided in Table S5.

Supporting Note 8. MSD of Monomer. We computed the MSDs of monomers and found similar trends to those of carbonyl oxygens, though the magnitudes are slightly smaller (Figure S8a). We attempted a theoretical Rouse-type prediction to fit the computed MSD of the monomers using a modified expression:

$$MSD = at^{\beta} + \frac{2\langle R_e^2\rangle}{\pi^2}\sum_{p=1}^{N-1}\frac{1-\exp\left(-p^2t/\tau_R\right)}{p^2} \tag{S13}$$

where the shorter time behavior of MSD is proportional to t^{β} and long-time behavior results from the collective Rouse relaxation of N − 1 eigenmodes associated with the COM positions of the monomers ($\mathrm{r}_i(t)$) of the polymer chain consisting of N beads.[16] $\langle R_e^2\rangle$ is the mean square end-to-end separation of the polymer chains. The Rouse mode analysis enables us to quantify the timescales associated with segmental motion in terms of MSD of monomers.

Supporting Note 9. MD Simulated Relaxation Kinetics around O–C–C–O Dihedral. As two adjacent EC units in a PEC chain are connected by an O–C–C–O dihedral, we have also investigated the relaxation kinetics around this dihedral by computing the following time correlation function (TCF)

$$C(t) = \langle \cos \delta\phi(t) \rangle \tag{S14}$$

where $\delta\phi(t)$ is the difference in dihedral angle around a given dihedral in the time interval t. Such a TCF enables direct estimation of the timescale for the relative motion of the first and fourth atoms about that dihedral. The TCFs were then fitted by using a sum of bi-exponential terms corresponding to shorter time (τ_{short}) and longer time (τ_{long}) behavior having a stretch component (β) using the following equation

$$C(t) = a\exp\left(-\frac{t}{\tau_{short}}\right) + (1-a)\exp\left(-\frac{t}{\tau_{long}}\right)^{\beta} \tag{S15}$$

Figure S8b presents the computed TCFs and accompanying fits for the motion around the O–C–C–O dihedral across the salt concentration range at 400 K. The fit parameters are tabulated in Table S7. From Figure S8b and Table S7, one can observe that the computed TCF for pure PEC exhibits the fastest long-time decay component, similar to the fastest MSD of its carbonyl oxygen. Upon addition of LiSCN, this dihedral relaxation becomes gradually slower up to 20 mol% of salt concentration. However, from the 40 mol% LiSCN system, the longer time component associated with the TCFs starts to decrease gradually. One can notice that from 60 mol% salt concentration onward, this dihedral relaxation becomes even faster than the 10 mol% system. From these combined analyses of MSDs of the carbonyl oxygen and torsional relaxation kinetics of the O–C–C–O dihedral, we can see that at higher LiSCN mol%, the polymer segmental motion becomes faster, in accordance to the experimental findings from DRS measurements.

Supporting Note 10. Calculation of Carbonate Coordination Number (CN) Surrounding Li-ion. In LiSCN-PEC SPEs, the carbonate monomer unit of PEC coordinates with Li-ion. The carbonate CN in the first coordination shell of Li-ion has been determined from the integrated area of the C=O stretch band according to the following equation:[17]

$$\mathrm{CN} = \frac{C_{\mathrm{bound}}}{C_{\mathrm{LiSCN}}} = \frac{C_{\mathrm{bound}}}{C_{\mathrm{total}}} \times \frac{C_{\mathrm{total}}}{C_{\mathrm{LiSCN}}} = \frac{C_{\mathrm{bound}}}{(C_{\mathrm{bound}}+C_{\mathrm{free}})} \times \frac{C_{\mathrm{total}}}{C_{\mathrm{LiSCN}}} \tag{S16}$$

Here C_{bound}, C_{free} and C_{total} is the Li-bound, free, and total carbonate concentrations, respectively. C_{LiSCN} is the LiSCN concentration. The integrated area (A_m) for bound or free C=O stretch band can be defined as: $A_m = \epsilon_m C_m L$ (m: bound or free), ϵ_m and L are the absorption coefficient associated with each C=O stretch mode and the path length of the IR cell, respectively. The relative integrated

area for Li^+-bound and free C=O stretch band is summarized in Table S8. Equation S16 can be further expressed in terms of A_m instead of C_m as

$$\mathrm{CN} = \frac{A_{\mathrm{bound}}}{\left(A_{\mathrm{bound}}+\frac{\epsilon_b}{\epsilon_f}A_{\mathrm{free}}\right)} \times \frac{C_{\mathrm{total}}}{C_{\mathrm{LiSCN}}} \quad \text{(S17)}$$

Lim et al. calculated $\frac{\epsilon_b}{\epsilon_f}$ ratio efficiently in a few linear and nonlinear carbonates by FT-IR measurements.[17] The carbonyl stretching IR spectra of linear carbonate like dimethyl carbonate (DMC) or diethyl carbonate (DEC) free from any complexity arises because of other contributions such as fermi resonance as observed in cyclic carbonates, e.g., propylene carbonate (PC). Henceforth the calculated $\frac{\epsilon_b}{\epsilon_f}$ ratio in DMC or DEC is more accurate than that of PC. In our present CN calculation, we used $\frac{\epsilon_b}{\epsilon_f}$ value of DMC that is 1.391.[17]

Supporting Note 11. C≡N Stretching Band in LiSCN-PC (or DMC) Solutions. Propylene carbonate (PC) solvent has high Li-salt solubility owing to its high dielectric constant (at room temperature ε_s ~ 65[18]). Therefore, free SCN- is expected to be present in a diluted solution. FTIR spectra have been recorded for diluted to concentrated solutions of LiSCN in PC. The LiSCN concentration-dependent CN stretching spectra in LiSCN-PC are shown in Figure S13a. Representative deconvoluted spectra in 1 and 10 mol% LiSCN-PC is shown in Figure S13b-c. With increasing LiSCN concentration, the absorbance intensity of the peak at 2074 cm^{-1} increases. The peak at 2063 cm^{-1} observed in the sppectrum of 1 mol% is absent in that off 10 mol% instead a new red-shifted peak emerges at 2052 cm^{-1}. In a diluted solution of 1 mol%, the 2063 cm^{-1} peak can be assigned to free SCN^-. As LiSCN concentration increases to 10 mol%, the signature of free SCN^-, 2063 cm^{-1} peak, disappears. Significant enhancement of 2074 cm^{-1} peak intensity from 1 to 10 mol% manifests in increased contact ion pairs while 2052 cm^{-1} may originate from complex ionic aggregates.

On the other hand, the dielectric constant of dimethyl carbonate (DMC) solvent is relatively closer to PEC (ε_s of DMC ~ 3.2[19] and PEC ~ 4[3b]). Therefore, we further compare the SCN^- stretch band in LiSCN-PEC with LiSCN-DMC mixtures. Three CN stretch peaks observed in LiSCN-DMC liquid mixture are very close to LiSCN-PEC SPE films where the same salt concentration is considered in both cases. LiSCN concentration dependent CN^--stretching FTIR spectra and relative population of each peak in LiSCN-DMC liquid mixture is shown in Figure S14.

Supporting Note 12. Simulation of Vibrational Spectra of SCN^-. We simulated the vibrational spectra of SCN^- anions across the entire salt concentration range (10 mol% to 100 mol%). We first extracted ~1000 $SCN(Li)_n$ clusters from the MD snapshots at each LiSCN mol% while keeping the relative populations of different $SCN(Li)_n$ clusters consistent with observations from Figure 4c. Each of the $SCN(LI)_n$ clusters was treated at the DFT level[20] using B3LYP/6-311++G(D,P) basis set, while rest of the bath atoms were incorporated as fixed molecular mechanics background charges. Subsequent geometry optimizations and harmonic vibrational frequency calculations for the SCN^- nuclear positions, while keeping the Li^+ nuclear coordinates frozen to sample all the possible geometries of the $SCN(Li)_n$ clusters, were performed with the Gaussian 16 package.[21] The optimization calculations converged for 90% of the total number of clusters. A frequency scaling factor of 0.967, consistent with the basis set, was used.

The transition dipole moment-weighted inhomogeneous frequency distribution was constructed to get the intensity profile of all the clusters and for clusters of a given type using the following equation:

$$I(\omega) = \langle \mu^2 \delta(\omega - \omega_0) \rangle \quad \text{(S18)}$$

Finally, we employed a Gaussian interpolation method on the obtained frequency distribution to get the final spectra.

Supporting Note 13. Pump-probe Infrared Measurements: Impact of LiSCN Concentration on Vibrational Lifetime of SCN^-. Pump-probe infrared measurements have been performed to understand the interaction of SCN^- in its first coordination shell with increasing salt concentration. Figure S18a-d shows the contour plot of the isotropic pump-probe signal of the C≡N stretch mode of SCN^- in LiSCN-PEC with varying salt concentrations. The red peaks have positive amplitudes, and they arise from the fundamental vibrational transitions originating from the ground-state bleach (GSB, $\upsilon = 0 \rightarrow 1$) and stimulated emission (SE, $\upsilon = 1 \rightarrow 0$) contributions. The blue peaks are negative, result from the excited-state absorption (ESA, $\upsilon = 1 \rightarrow 2$), and are red-shifted from the red peaks due to vibrational anharmonicities.[22] Corresponding transient pump-probe spectra with different pump-probe delay times is shown in Figure S18e-h. Note that the positive and negative signals for an individual peak of C≡N stretch are not clearly observed in the pump-probe signal. This is because of destructive interference due to partial or complete overlapping of one positive signal with another negative

signal. The vibrational population relaxation time (τ_{life}) has been determined from the population growth/decay of $1 \rightarrow 2$ transition corresponding to the 2042 cm^{-1} peak and $0 \rightarrow 1$ transition of the 2096 cm^{-1} peak. Note we consider four consecutive frequencies positioned at the red end of $1 \rightarrow 2$ transition peak corresponding to the 2042 cm^{-1} peak and the blue end of $0 \rightarrow 1$ transition of the 2096 cm^{-1} peak to determine the τ_{life}. Each population decay is well described by an exponential function, represented in Figure S18i-l and τ_{life} is the average of four frequencies considered for each peak. The observed τ_{life} values are summarized in Table S9. The τ_{life} associated with $1 \rightarrow 2$ transition of 2042 cm^{-1} peak lengthens with increasing LiSCN concentration, while for $0 \rightarrow 1$ transition of 2096 cm^{-1} peak, it remains nearly unchanged. The variation of $\langle\tau_{life}\rangle$ for $1 \rightarrow 2$ transition of 2042 cm^{-1} peak indicates that this peak is composed of two different solvation species, which cannot be resolved in the frequency domain.

Table S1. DC ionic condutivity (σ_{dc}, S cm^{-1}) at various temperature and activation energies ($E_a^{\sigma_{\text{dc}}}$, kJ mol^{-1}) in x mol% LiSCN-PEC SPE films. σ_{dc} shown in parentheses were determined from equivalent circuit fit of respective complex impedance spectra.

mol% LiSCN	σ_{dc}						$E_a^{\sigma_{\text{dc}}}$
	298 K	303 K	308 K	313 K	318 K	323 K	
10	$5.28\text{x}10^{-9}$ ($5.21\text{x}10^{-9}$)	$1.04\text{x}10^{-8}$ ($1.01\text{x}10^{-8}$)	$1.91\text{x}10^{-8}$ ($1.88\text{x}10^{-8}$)	$3.56\text{x}10^{-8}$ ($3.56\text{x}10^{-8}$)	$7.01\text{x}10^{-8}$ ($7.10\text{x}10^{-8}$)	$1.10\text{x}10^{-7}$ ($1.06\text{x}10^{-7}$)	97.60
20	$6.50\text{x}10^{-9}$ ($6.43\text{x}10^{-9}$)	$1.27\text{x}10^{-8}$ ($1.25\text{x}10^{-8}$)	$2.41\text{x}10^{-8}$ ($2.35\text{x}10^{-8}$)	$4.30\text{x}10^{-8}$ ($4.24\text{x}10^{-8}$)	$7.84\text{x}10^{-8}$ ($7.71\text{x}10^{-8}$)	$1.37\text{x}10^{-7}$ ($1.36\text{x}10^{-7}$)	98.14
40	$2.78\text{x}10^{-7}$ ($2.76\text{x}10^{-7}$)	$4.82\text{x}10^{-7}$ ($4.78\text{x}10^{-7}$)	$8.38\text{x}10^{-7}$ ($8.30\text{x}10^{-7}$)	$1.44\text{x}10^{-6}$ ($1.44\text{x}10^{-6}$)	$2.47\text{x}10^{-6}$ ($2.49\text{x}10^{-6}$)	$4.30\text{x}10^{-6}$ ($4.30\text{x}10^{-6}$)	87.85
60	$6.83\text{x}10^{-7}$ ($6.80\text{x}10^{-7}$)	$1.24\text{x}10^{-6}$ ($1.24\text{x}10^{-6}$)	$2.16\text{x}10^{-6}$ ($2.17\text{x}10^{-6}$)	$3.73\text{x}10^{-6}$ ($3.76\text{x}10^{-6}$)	$6.19\text{x}10^{-6}$ ($6.26\text{x}10^{-6}$)	$1.04\text{x}10^{-5}$ ($1.06\text{x}10^{-5}$)	87.15
80	$1.58\text{x}10^{-6}$ ($1.56\text{x}10^{-6}$)	$2.88\text{x}10^{-6}$ ($2.85\text{x}10^{-6}$)	$4.66\text{x}10^{-6}$ ($4.54\text{x}10^{-6}$)	$7.62\text{x}10^{-6}$ ($7.46\text{x}10^{-6}$)	$1.38\text{x}10^{-5}$ ($1.37\text{x}10^{-5}$)	$2.15\text{x}10^{-5}$ ($2.09\text{x}10^{-5}$)	83.90
100	$1.52\text{x}10^{-6}$ ($1.50\text{x}10^{-6}$)	$2.83\text{x}10^{-6}$ ($2.82\text{x}10^{-6}$)	$5.08\text{x}10^{-6}$ ($5.10\text{x}10^{-6}$)	$9.02\text{x}10^{-6}$ ($8.99\text{x}10^{-6}$)	$1.50\text{x}10^{-5}$ ($1.50\text{x}10^{-5}$)	$3.16\text{x}10^{-5}$ ($3.13\text{x}10^{-5}$)	94.93

Table S2. Decomposition of average binding energy for LiFSI-PEC and LiTFSI-PEC SPEs

mol% LiFSI	$E_{BE}^{Li^+}$ (kJmol^{-1})		
	Total	Ionic	Polymer
10%	-138.522	-71.0648	-67.457
20%	-138.088	-79.8575	-58.230
40%	-137.858	-94.4689	-43.389
60%	-136.09	-102.017	-34.073
80%	-136.529	-108.74	-27.789
100%	-134.297	-111.081	-23.216
mol% LiTFSI	$E_{BE}^{Li^+}$ (kJmol^{-1})		
	Total	Ionic	Polymer
10%	-134.047	-53.2123	-80.835
20%	-136.557	-65.938	-65.938
40%	-135.517	-79.1196	-56.398
60%	-133.004	-89.0129	-43.991
80%	-129.517	-94.2982	-35.219
100%	-130.619	-97.4856	-33.133

Table S3. Polymer segmental relaxation times (τ_s) by DRS studies in LiSCN-PEC SPE films at various temperatures

mol% LiSCN	τ_s (µs)					
	298 K	303 K	308 K	313 K	318 K	323 K
10	184.65	62.01	32.60	16.99	8.52	4.97
20	126.75	67.56	31.11	19.59	9.24	6.41
40	13.87	8.60	5.49	2.92	1.54	0.96
60	9.22	4.68	2.59	1.53	0.88	0.52
80	5.20	3.05	1.92	1.08	0.53	0.33

100	7.41	4.10	2.30	1.13	0.70	0.35

Table S4. The values of thickness (d), area (A), volume (V) and molar concentration (c) for x mol% LiSCN-PEC SPE films.

mol% LiSCN	d (cm)	A (cm^2)	V (cm^3)	mol of LiSCN (x 10^{-3})	c (molcm^{-3}) (x 10^{-3})
10	0.0084	2.01	0.0169	0.01223	0.72367
20	0.0075	2.01	0.0151	0.02210	1.46358
40	0.0135	2.01	0.0271	0.05880	2.16974
60	0.0125	2.01	0.0251	0.08427	3.35737
80	0.0118	2.01	0.0237	0.09960	4.20253
100	0.0132	2.01	0.0265	0.13253	5.00113

Table S5. Molar ionic conductivity (Λ, Scm^{2}mol^{-1}) of x mol% LiSCN-PEC SPE films at various temperature (T, K).

mol% LiSCN	Λ					
	298 K	303 K	308 K	313 K	318 K	323 K
10	7.30x10^{-6}	1.44x10^{-5}	2.64x10^{-5}	4.92x10^{-5}	9.69x10^{-5}	1.52x10^{-4}
20	4.44x10^{-6}	8.70x10^{-6}	1.65x10^{-5}	2.93x10^{-5}	5.36x10^{-5}	9.35x10^{-5}
40	1.28x10^{-4}	2.22x10^{-4}	3.86x10^{-4}	6.66x10^{-4}	1.14x10^{-3}	1.98x10^{-3}
60	2.03x10^{-4}	3.69x10^{-4}	6.43x10^{-4}	1.11x10^{-3}	1.84x10^{-3}	3.10x10^{-3}
80	3.76x10^{-4}	6.84x10^{-4}	1.11x10^{-3}	1.81x10^{-3}	3.28x10^{-3}	5.12x10^{-3}
100	3.03x10^{-4}	5.67x10^{-4}	1.02x10^{-3}	1.80x10^{-3}	3.00x10^{-3}	6.32x10^{-3}

Table S6. Computed diffusion coefficients (D) of carbonyl oxygens and Rouse model fit parameters for MSDs of monomer at 400 K.

LiSCN mol%	D (x10^{-12}, m^{2}s^{-1}) of C=O	Rouse model fit parameters for MSDs of EC			
		a	β	$\langle R_e^2 \rangle$ (Å^2)	τ_R (ms)
0	4.97	2.09	0.23	23770.20	3.76
10	1.53	1.36	0.14	22073.20	15.18
20	1.10	1.13	0.16	11081.20	16.24
40	1.23	1.07	0.15	8966.01	7.93
60	1.96	1.22	0.22	12388.70	6.83
80	2.45	1.40	0.23	8639.41	3.22
100	3.75	1.51	0.24	15017.70	3.24

Table S7. The fit parameters corresponding to O–C–C–O dihedral relaxation at 400 K.

LiSCN mol%	a	τ_{short} (ns)	τ_{long} (ns)	β
0	0.136	0.044	16.108	0.27
10	0.117	0.054	174.478	0.22
20	0.111	0.060	574.732	0.20
40	0.068	0.037	378.273	0.21
60	0.049	0.040	135.646	0.23
80	0.059	0.114	129.770	0.23
100	0.047	0.187	69.425	0.24

Table S8. The relative integrated area for Li^+-bound (A_{bound}) and free C=O stretch band (A_{free}), relative mol number of carbonate (C_{total}), LiSCN (C_{LiSCN}) and the carbonate coordination number in first solvation shell of Li-ion (CN) in x mol% LiSCN-PEC SPE films.

x mol% of LiSCN	A_{bound}(%)	A_{free} (%)	C_{total} (mol)	C_{LiSCN} (mol)	CN
10	30	70	100	10	2.4
20	51	49	100	20	2.1
40	62	38	100	40	1.4
60	65	35	100	60	1.0
80	79	21	100	80	0.9
100	87	13	100	100	0.8

Table S9. Excited state vibrational lifetime of C≡N stretch mode in different SCN^- species present in LiSCN-PEC SPEs

LiSCN mol%	τ_{life} of 2042 cm^{-1} species (1 → 2 transition) (ps)	τ_{life} of 2096 cm^{-1} species (0 → 1 transition) (ps)
10	16	30
20	17	24
40	22	32
60	27	31

Scheme S1: Chemical structures of (a) polyethylene carbonate (PEC), (b) lithium thiocyanate (LiSCN) and (c) monomer of PEC.

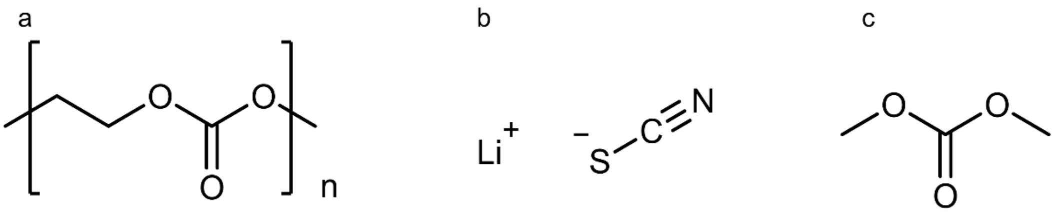

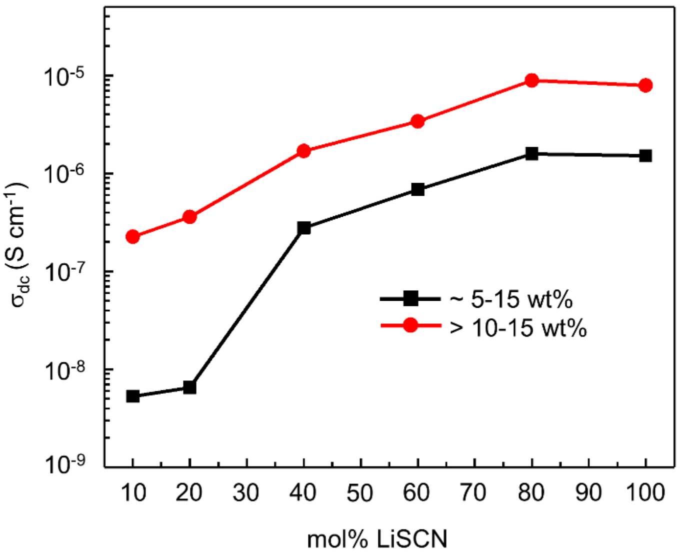

Figure S1. Residual solvent impact. Impact of residual solvent on ionic conductivity of LiSCN-PEC SPEs as a function of LiSCN concentration. The black symbols indicate residual solvent content in the SPEs are within 5-15 wt%, while red symbols indicate the residual solvent content is > 10-15 wt%.

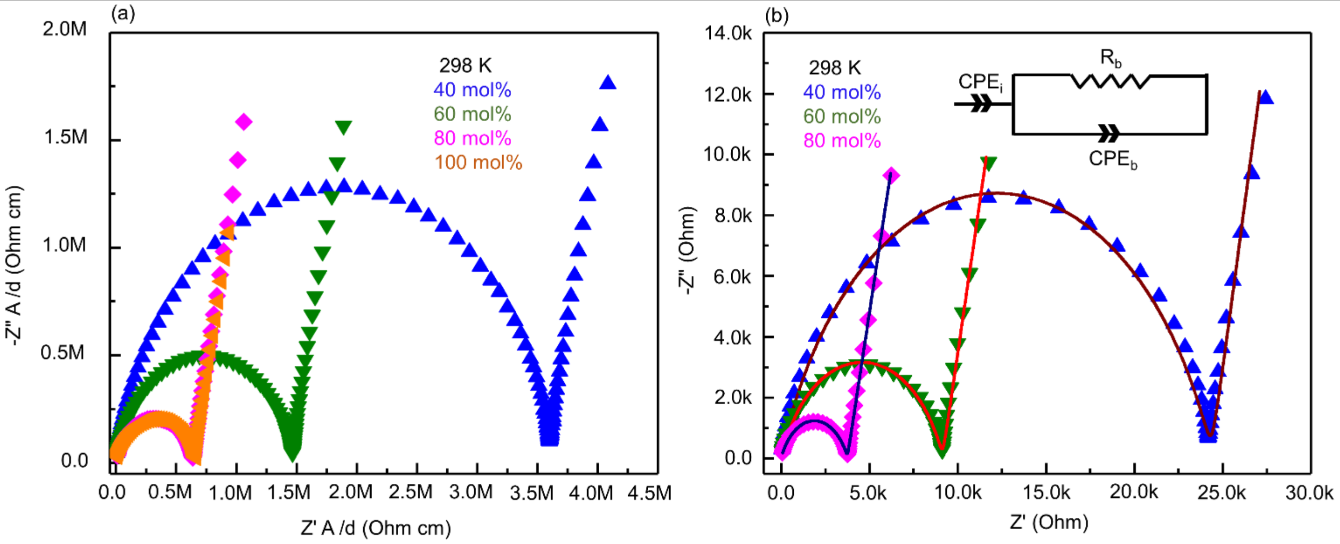

Fig. S2. Representative impedance spectra in LiSCN-PEC SPEs in Nyquist presentation. (a) To account for the varying film thicknesses of each SPE, impedance data were normalized by multiplying with the factor *A*/*d*, (where *A* and *d* are the film area and thickness, respectively) enabling direct comparison of the bulk resistance irrespective of film thickness. (b) Raw impedance spectra fitted to an equivalent circuit model.

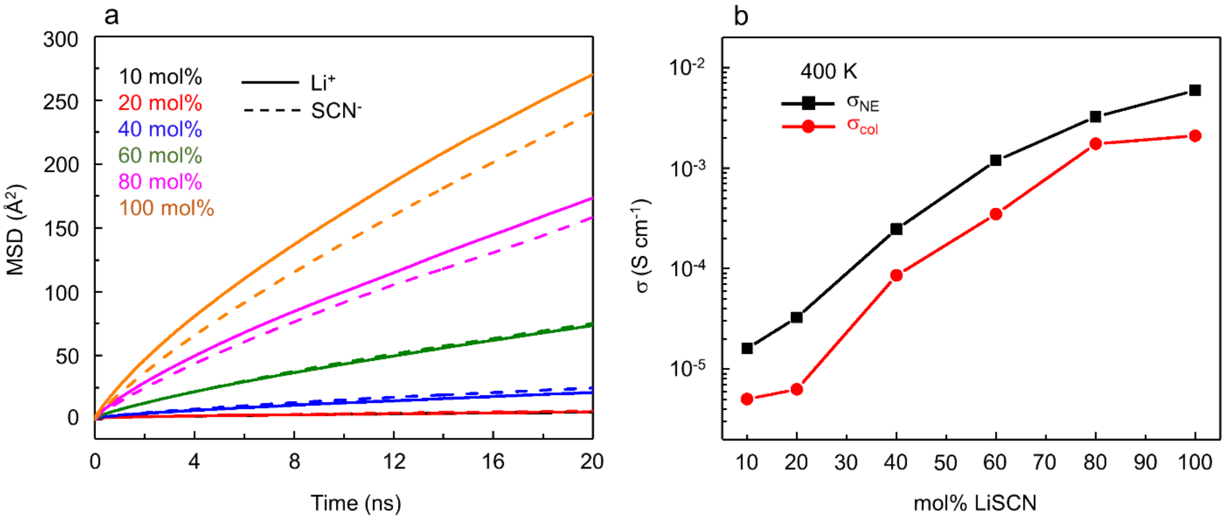

Figure S3. Simulated ion diffusion. (a) MSDs of Li^+ (solid lines) and SCN^- (dashed lines) (b) Nernst-Einstein (σ_{NE}) and collective (σ_{col}) ionic conductivities as a function of LiSCN concentrations at 400 K.

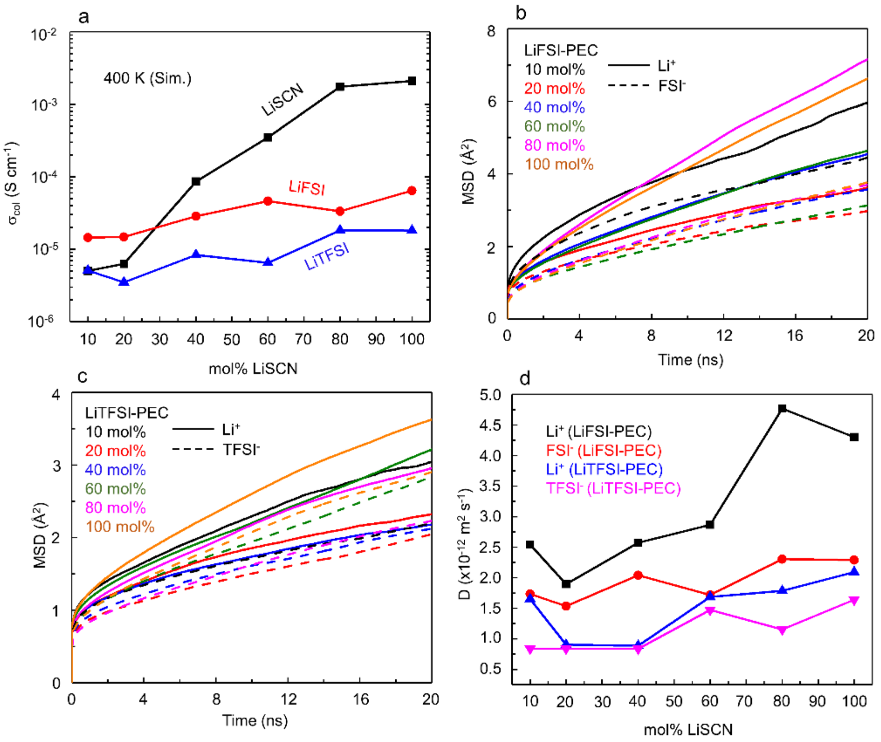

Figure S4. Anion impact on ion conductivity. (a) Comparison of MD simulated collective ionic conductivity between LiSCN-PEC, LiFSI-PEC, and LiTFSI-PEC SPEs as a function of salt concentration. (b) MSDs of Li^+ (solid lines) and FSI^- (dashed lines) in LiFSI-PEC SPEs, (c) MSDs of Li^+ (solid lines) and $TFSI^-$ (dashed lines) in LiTFSI-PEC SPEs and (d) translational self-diffusion coefficients of Li^+, FSI^- in LiFSI-PEC and Li^+, $TFSI^-$ ions in LiTFSI-PEC SPEs as a function of Li-salt concentrations at 400 K.

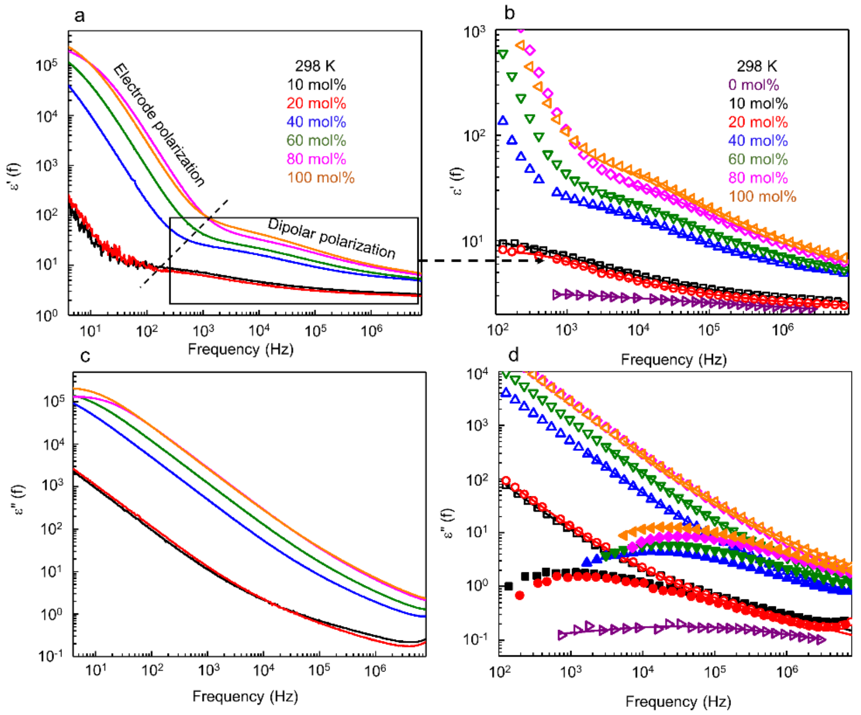

Figure S5. DRS analyses. (a) Real ($\varepsilon'(f)$) component of complex dielectric spectra in LiSCN-PEC SPEs as a function of frequency at a representative temperature 298 K. Different LiSCN concentrations are indicated in the inset with color code. The dashed line in $\varepsilon'(f)$ spectrum is a visual separation of dipolar polarization and electrode polarization contribution region. The dipolar contribution has been analyzed from the region shown in the rectangular box. (b) The solid lines passing through $\varepsilon'(f)$ are respective HN fits (eq. S8). (c) imaginary ($\varepsilon''(f)$) component of complex dielectric spectra. (d) The LiSCN concentration-dependent $\varepsilon''(f)$ (open symbols) at a representative temperature of 298 K. The solid lines passing through $\varepsilon''(f)$ are respective HN fits (eq. S8). The filled symbols represent the $\varepsilon''_{dipole}(f)$ due to dipolar reorientation polarization relaxation dynamics obtained after subtracting the DC conductivity contribution from the total as $\varepsilon''(f)$ $\varepsilon''_{dipole}(f) = \varepsilon''(f) - \varepsilon''_{\sigma_{dc}}(f)$. All are color-coded.

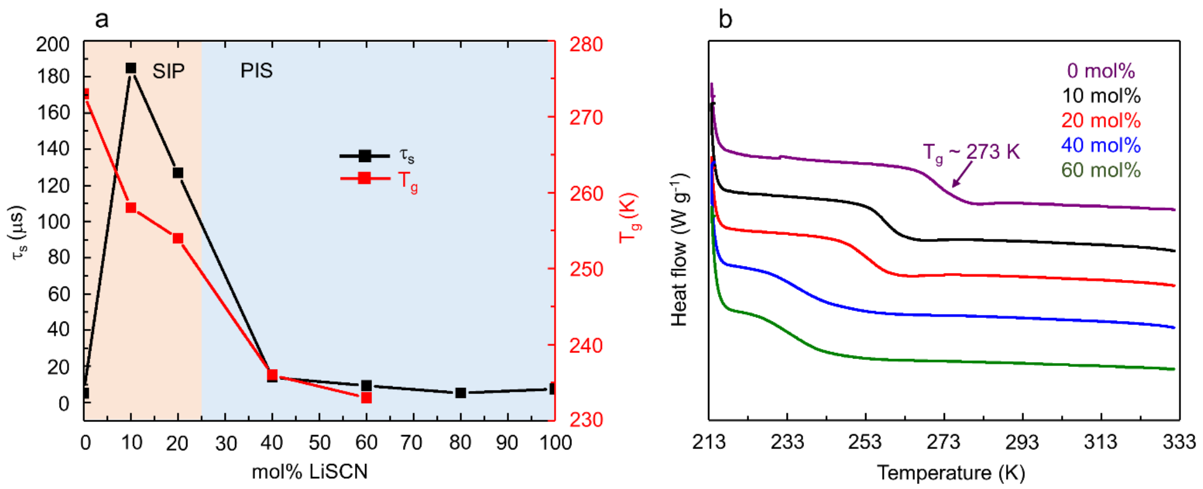

Figure S6. Glass transition temperature. (a) LiSCN concentration dependent τ_s (at 298 K) and T_g of the LiSCN-PEC SPE films. (b) DSC thermogram of LiSCN-PEC SPE films with different salt concentrations and pure PEC.

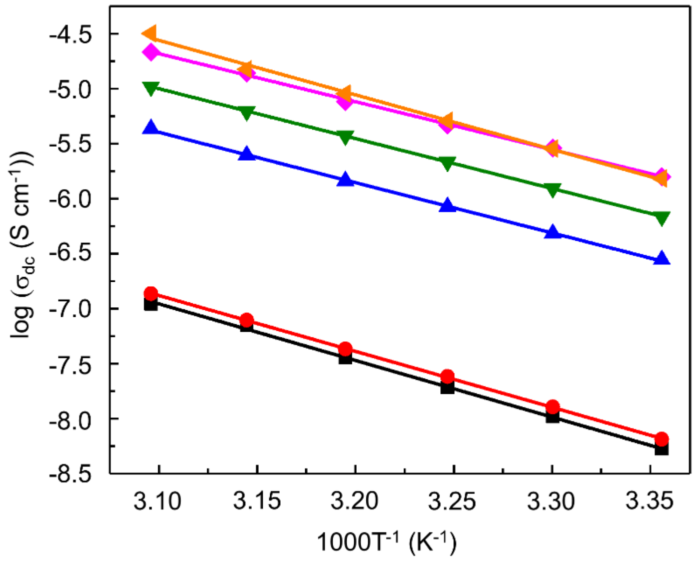

Figure S7. Activation energy. Arrhenius plot of experimental σ_{dc}.

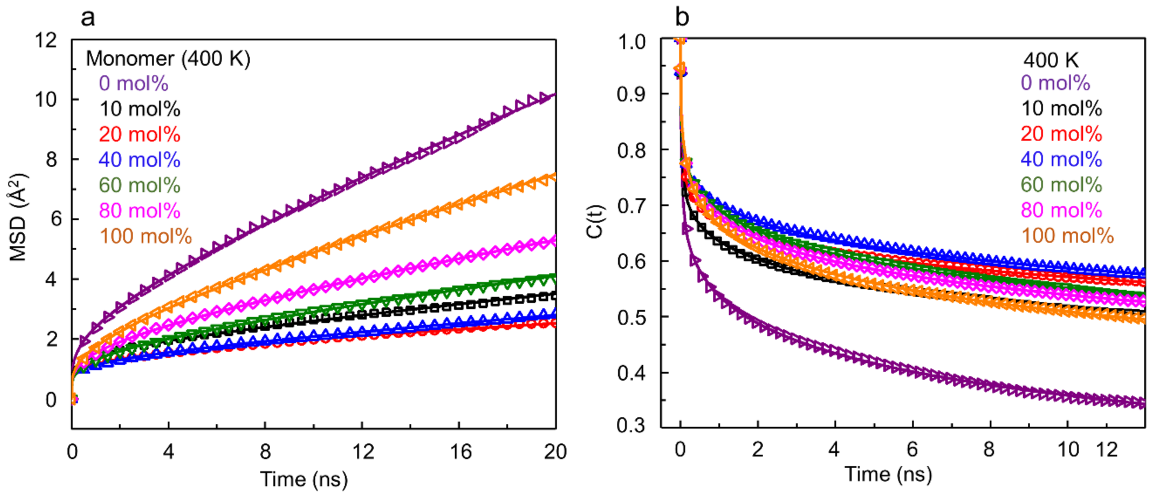

Figure S8. Simulated polymer segmental dynamics.(a) MSDs of EC monomer unit in the COM frame of the PEC chain as a function of LiSCN concentrations at 400 K. Open symbols are MSD data points, and solid lines passing through the MSD data points are fitted lines using Rouse-type model. (b) O–C–C–O dihedral relaxation as a function of LiSCN concentrations at 400 K.

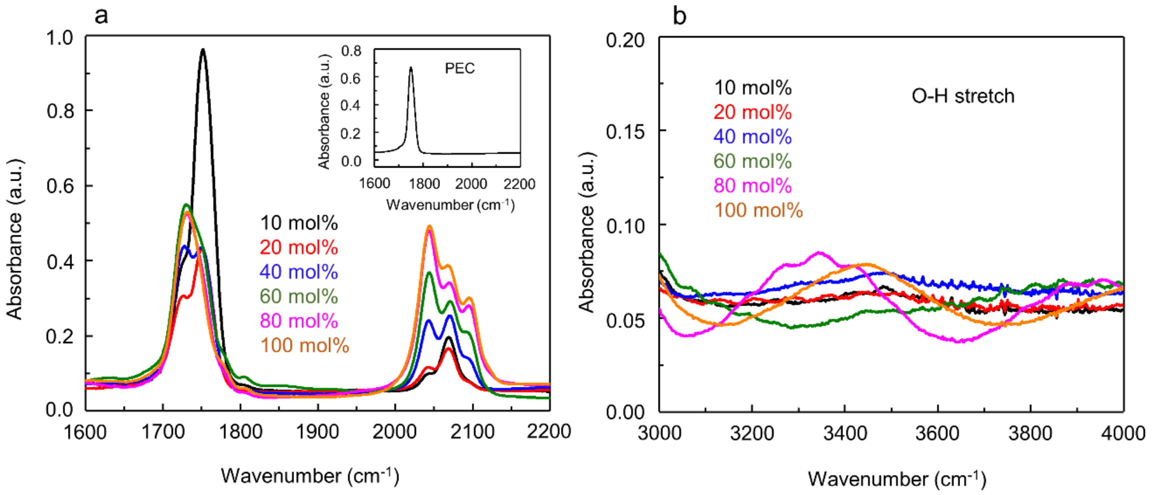

Figure S9. FTIR absorption spectra of LiSCN-PEC SPE films with different salt concentrations. (b) IR absorption spectrum around O-H stretching mode in those SPEs.

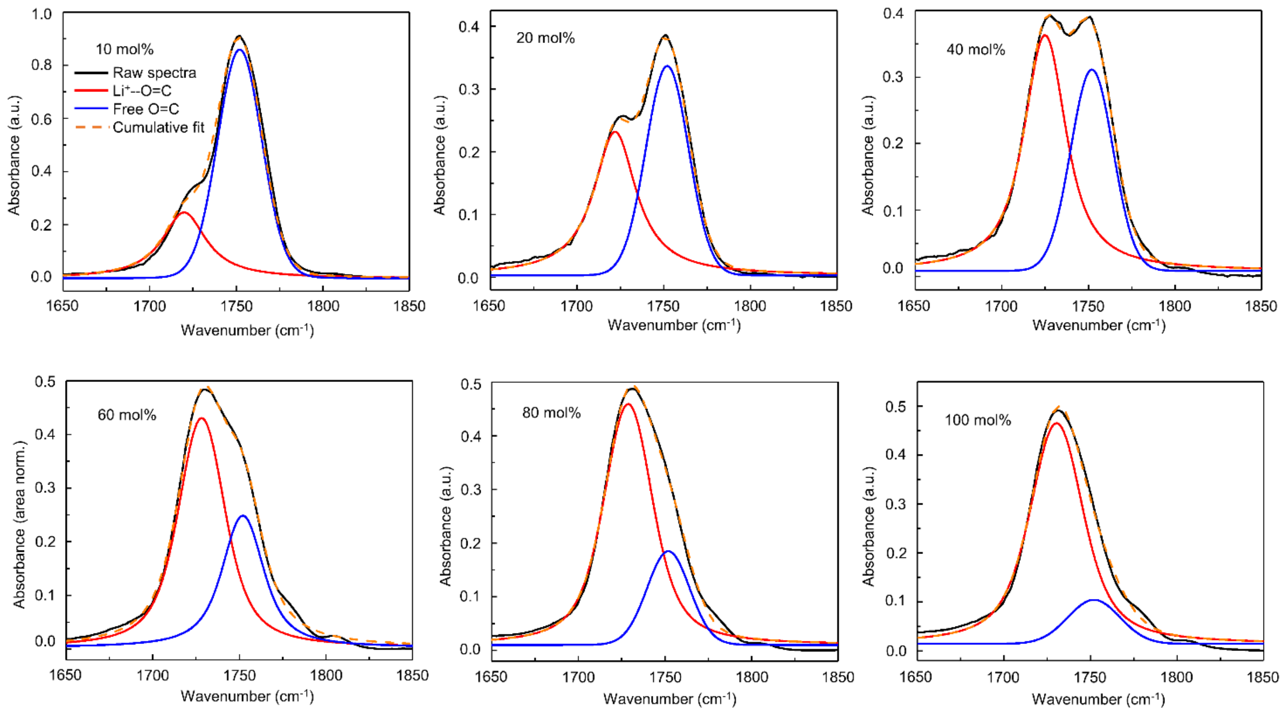

Figure S10. Deconvolution of raw FTIR absorption spectra of C=O stretch by two Voigt functions in LiSCN-PEC SPE films with different salt concentrations. LiSCN mol% in each plot is mentioned.

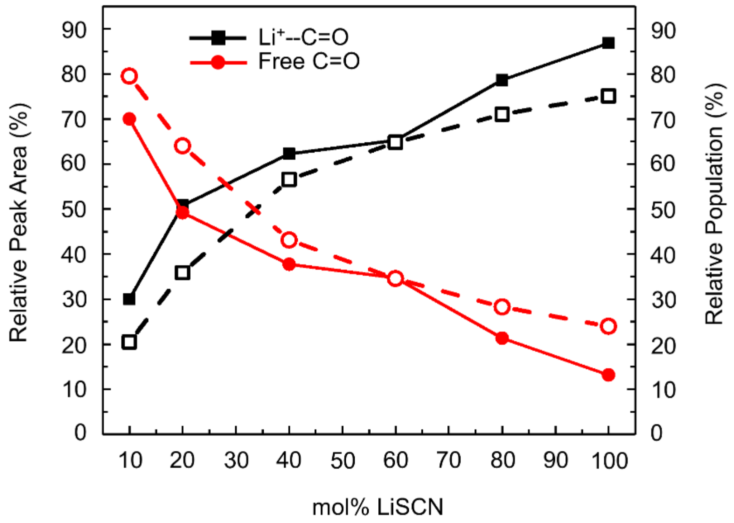

Figure S11. Relative peak area of free C=O and Li^+-bound C=O from experiment (filled symbols with solid lines) and relative population of free C=O and Li^+-bound C=O from simulation (open symbols with dashed lines) at different LiSCN mol%.

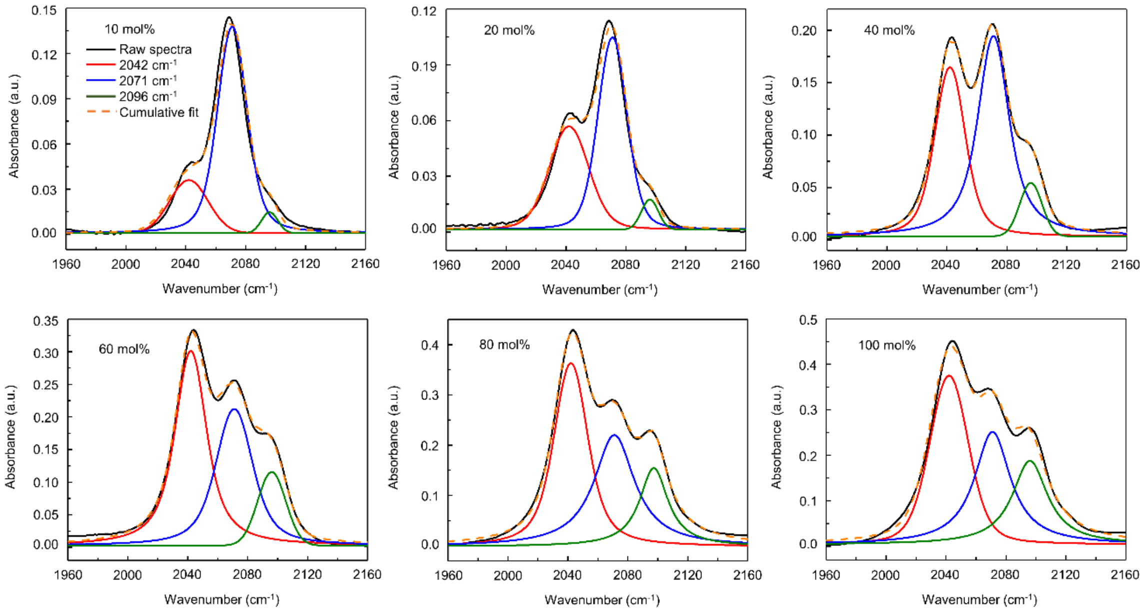

Figure S12. Deconvolution of raw FTIR absorption spectra of C≡N stretch by 3 Voigt functions in LiSCN-PEC SPE films with different salt concentrations. LiSCN mol% in each plot is mentioned.

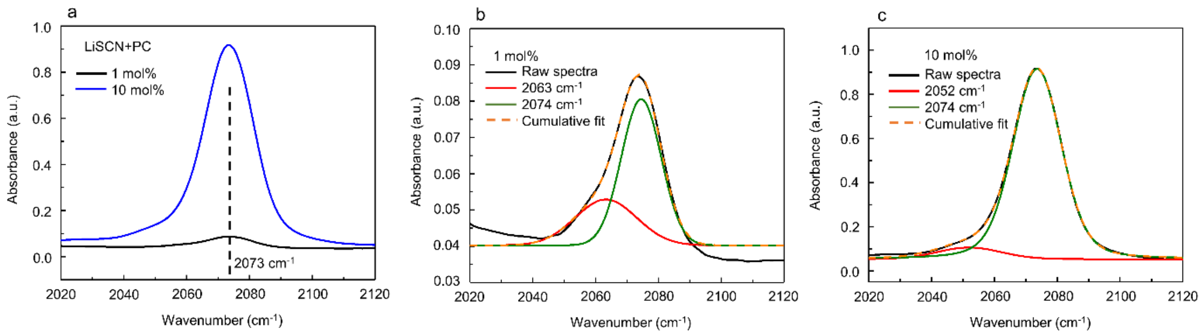

Figure S13. (a) FTIR absorption spectra of SCN^- stretching vibration in LiSCN-PC at different salt concentrations. (b-c) deconvolution of raw FTIR absorption spectra by Voigt functions at (b) 1 mol% and (c) 10 mol% salt concentration.

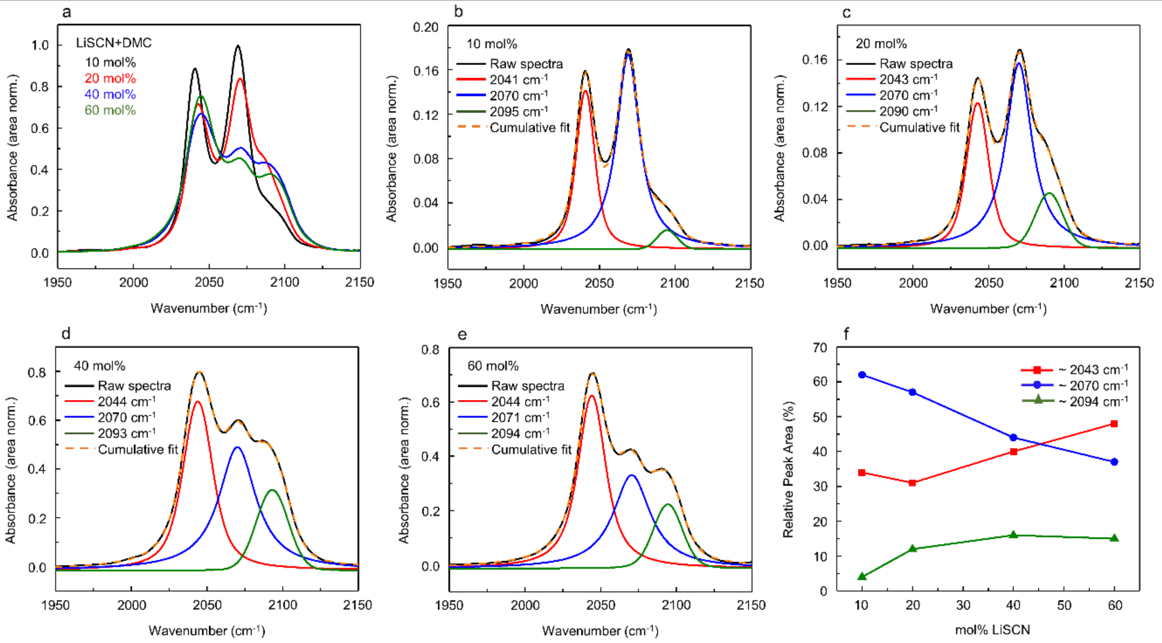

Figure S14. FTIR spectral analyses in LiSCN-DMC mixture: (a) Area normalized FTIR absorption spectra of SCN^- stretching vibration, (b-e) deconvolution of raw FTIR absorption spectra of C≡N stretch by 3 Voigt functions, (f) LiSCN mol% dependent variation of peak area contribution for each peak of SCN^- stretch band.

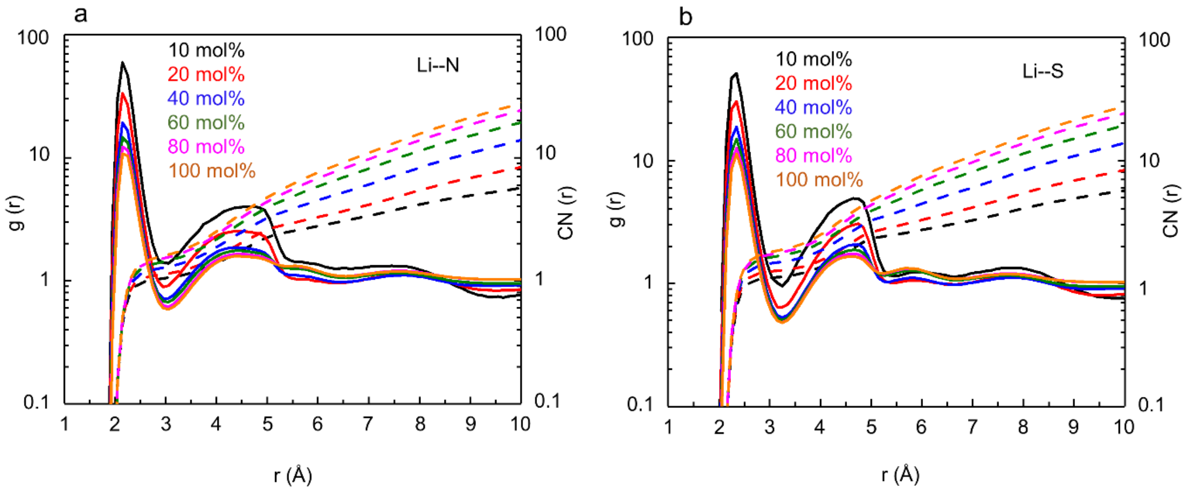

Figure S15: The radial distribution function ($g(r)$) and radial coordination number ($CN(r)$) between the (a) Li--N pair and (b) Li--S pair at 400 K. $g(r)$ and $CN(r)$ are shown as solid lines and dashed lines, respectively. All are color-coded.

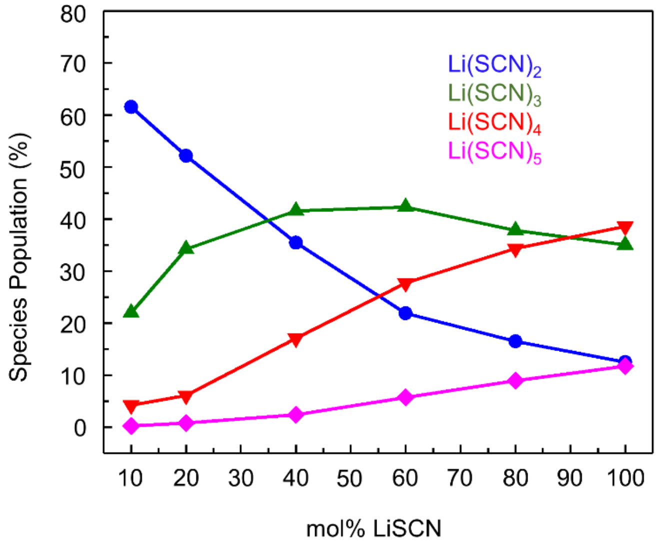

Figure S16. Solvation shell analysis. MD simulated population of $[Li(SCN)_n]^{(n-1)-}$ (n=2-5) in the first solvation shell at 400 K.

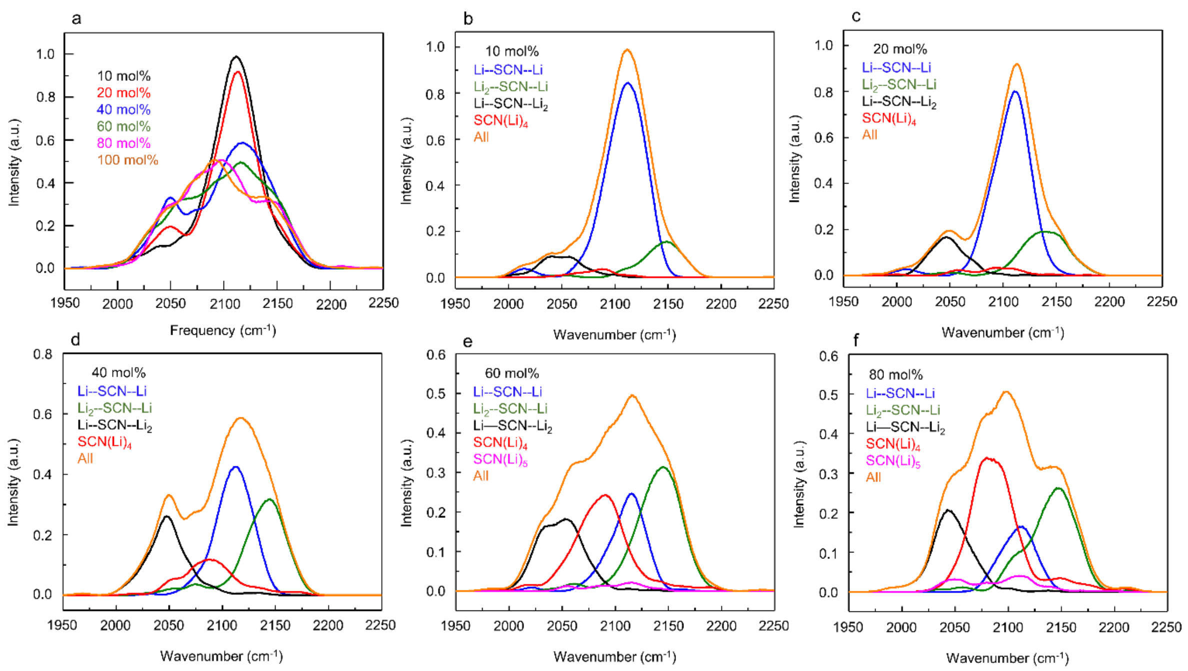

Figure S17. Simulated SCN- spectra. (a) Simulated spectra of SCN- ions at different salt concentrations. (b-f) Deconvolution of simulated SCN- spectra to various $[SCN(Li)_n]^{(n-1)+}$ (n= 2-5) clusters for different LiSCN mol%.

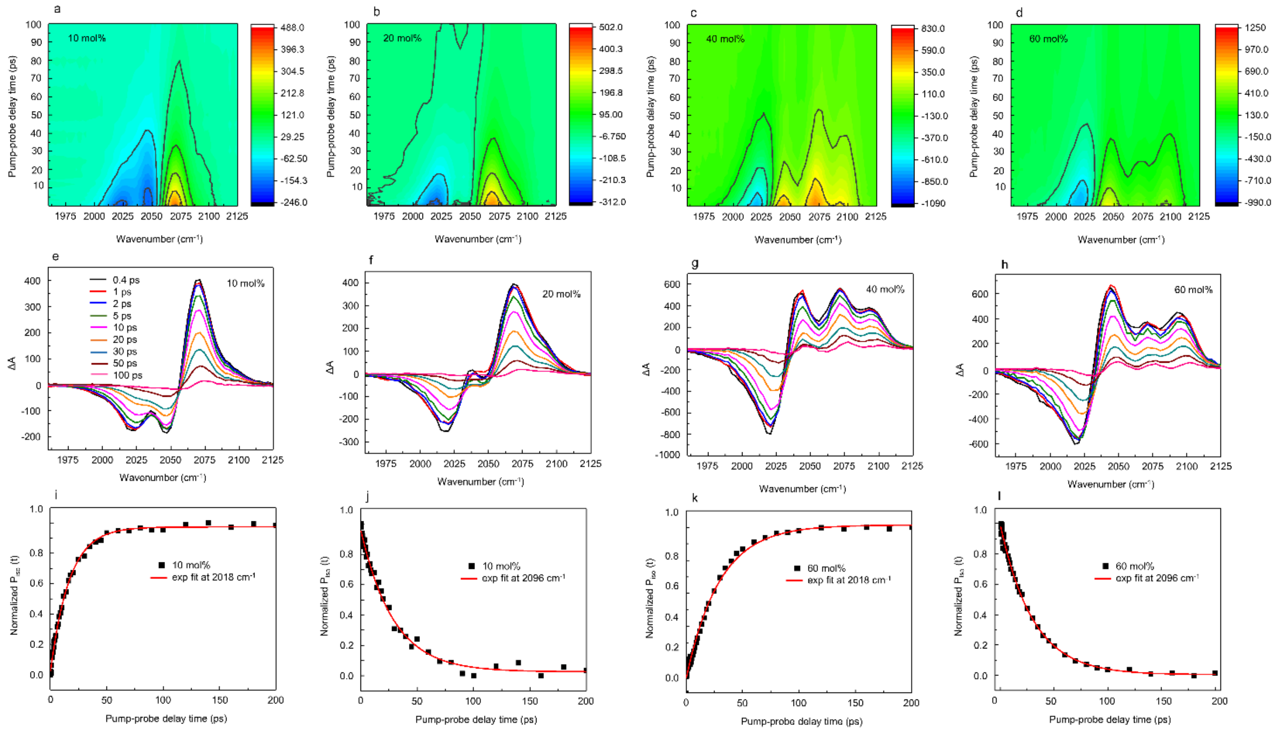

Figure S18. IR pump-probe spectra analysis in LiSCN-PEC SPEs. (a-d): Isotropic IR pump-probe signal of C≡N stretch mode of SCN^- in LiSCN-PEC with different salt concentrations. (e-h): Isotropic IR pump-probe spectra at different pump-probe delay times of C≡N stretch mode of SCN^- in LiSCN-PEC with different salt concentrations. (i-l): Representative isotropic vibrational population relaxation decays.

Supporting References

[1] C. Liu, R. L. Sacci, R. Sahore, G. M. Veith, N. J. Dudney, X. C. Chen, *J. Power Sources* **2022**, *527*, 231165.

[2] C. Tian, J. Tang, L. Wang, R. Huang, C. Ai, H. Cao, T. Huang, A. Yu, *ACS Sustain. Chem. Eng.* **2023**, *11*, 10164−10171.

[3] a) K. Kimura, J. Motomatsu, Y. Tominaga, *J. Phys. Chem. C* **2016**, *120*, 12385-12391; b) J. Motomatsu, H. Kodama, T. Furukawa, Y. Tominaga, *Macromol. Chem. Phys.* **2015**, *216*, 1660-1665; c) K. Kimura, J. Motomatsu, Y. Tominaga, *J. Polym. Sci., Part B: Polym. Phys.* **2016**, *54*, 2442-2447.

[4] a) K. Kumbhakar, T. D. Pham, K.-K. Lee, K. Kwak, M. Cho, *Electrochim. Acta* **2023**, *462*, 142759; b) F. Kremer, A. Schönhals, *Broadband dielectric spectroscopy*, Springer Science & Business Media, **2002**.

[5] a) S. Havriliak, S. Negami, *Polym.* **1967**, *8*, 161-210; b) S. Havriliak, S. Negami, in *Journal of Polymer Science Part C: Polymer Symposia, Vol. 14*, Wiley Online Library, **1966**, pp. 99-117.

[6] P. J. in't Veld, G. C. Rutledge, *Macromolecules* **2003**, *36*, 7358-7365.

[7] S. Plimpton, *J. Comput. Phys.* **1995**, *117*, 1-19.

[8] J.-C. Soetens, C. Millot, B. Maigret, *J. Phys. Chem. A* **1998**, *102*, 1055-1061.

[9] a) T. Hou, K. D. Fong, J. Wang, K. A. Persson, *Chem. Sci.* **2021**, *12*, 14740-14751; b) B. Doherty, X. Zhong, S. Gathiaka, B. Li, O. Acevedo, *J. Chem. Theory Comput.* **2017**, *13*, 6131-6145.

[10] J.-P. Ryckaert, G. Ciccotti, H. J. Berendsen, *J. Comput. Phys.* **1977**, *23*, 327-341.

[11] a) S. Nosé, *Mol. Phys.* **1984**, *52*, 255-268; b) W. G. Hoover, *Phys. Rev. A* **1985**, *31*, 1695-1697.

[12] B. A. Luty, W. F. van Gunsteren, *J. Phys. Chem.* **1996**, *100*, 2581-2587.

[13] W. Humphrey, A. Dalke, K. Schulten, *J. Mol. Graph.* **1996**, *14*, 33-38.

[14] H. K. Kashyap, H. V. R. Annapureddy, F. O. Raineri, C. J. Margulis, *J. Phys. Chem. B* **2011**, *115*, 13212-13221.

[15] a) A. France-Lanord, J. C. Grossman, *Phys. Rev. Lett.* **2019**, *122*, 136001; b) C. Y. Son, Z.-G. Wang, *J. Chem. Phys.* **2020**, *153*, 100903; c) F. Müller-Plathe, W. F. van Gunsteren, *J. Chem. Phys.* **1995**, *103*, 4745-4756.

[16] A. Maitra, A. Heuer, *Phys. Rev. Lett.* **2007**, *98*, 227802.

[17] C. Lim, J. H. Kim, Y. Chae, K.-K. Lee, K. Kwak, M. Cho, *Anal. Chem.* **2021**, *93*, 12594-12601.

[18] K. Mukherjee, K. Kumbhakar, R. Biswas, *J. Mol. Liq.* **2022**, *360*, 119491.

[19] R. Naejus, D. Lemordant, R. Coudert, P. Willmann, *J. Chem. Thermodyn.* **1997**, *29*, 1503-1515.

[20] a) W. Kohn, L. J. Sham, *Phys. Rev.* **1965**, *140*, A1133-A1138; b) J. Patterson, S. Lehoczky, *Phys. Lett. A* **1989**, *137*, 137-138.

[21] M. e. Frisch, G. Trucks, H. Schlegel, G. Scuseria, M. Robb, J. Cheeseman, G. Scalmani, V. Barone, G. Petersson, H. Nakatsuji, Gaussian, Inc., Wallingford CT, **2016**.

[22] a) K.-K. Lee, K.-H. Park, D. Kwon, J.-H. Choi, H. Son, S. Park, M. Cho, *J. Chem. Phys.* **2011**, *134*, 064506; b) S. Mondal, J. Kang, K. Park, J. M. Lim, J.-H. Ha, K. Kwak, M. Cho, *J. Phys. Chem. Lett.* **2021**, *12*, 9275-9282; c) J. Lim, K.-K. Lee, C. Liang, K.-H. Park, M. Kim, K. Kwak, M. Cho, *J. Phys. Chem. B* **2019**, *123*, 6651-6663; d) J. Lim, K. Park, H. Lee, J. Kim, K. Kwak, M. Cho, *J. Am. Chem. Soc.* **2018**, *140*, 15661-15667.
